# Mapping the incidence rate of typhoid fever in sub-Saharan Africa

**DOI:** 10.1371/journal.pntd.0011902

**Published:** 2024-02-26

**Authors:** Jong-Hoon Kim, Jungsoon Choi, Chaelin Kim, Gi Deok Pak, Prerana Parajulee, Andrea Haselbeck, Se Eun Park, Vittal Mogasale, Hyon Jin Jeon, Annie J. Browne, Ellis Owusu-Dabo, Raphaël Rakotozandrindrainy, Abdramane Soura Bassiahi, Mekonnen Teferi, Octavie Lunguya-Metila, Christiane Dolecek, Virginia E. Pitzer, John A. Crump, Simon I. Hay, Florian Marks

**Affiliations:** 1 International Vaccine Institute, Seoul, Republic of Korea; 2 Department of Mathematics, Hanyang University, Seoul, Republic of Korea; 3 Graduate School of Public Health, Yonsei University, Seoul, Republic of Korea; 4 International Vaccine Institute, Seoul, Republic of Korea; 5 Cambridge Institute of Therapeutic Immunology and Infectious Disease, University of Cambridge School of Clinical Medicine, Cambridge Biomedical Campus, Cambridge, United Kingdom; 6 Madagascar Institute for Vaccine Research, University of Antananarivo, Antananarivo, Madagascar; 7 Malaria Atlas Project, Telethon Kids Institute, Perth, Australia; 8 School of Public Health, Kwame Nkrumah University of Science and Technology, Laing Building Complex J.W. Acheampong CI, Kumasi, Ghana; 9 Institut Supérieur des Sciences de la Population, Ouagadougou, Burkina Faso; 10 Armauer Hansen Research Institute, ALERT Compound Zenebework, Addis Ababa, Ethiopia; 11 Department of Microbiology, Institut National de Recherche Biomédicale, Kinshasa, Democratic Republic of Congo; 12 Department of Medical Biology, Microbiology Service, University Teaching Hospital, Ave De L’hopital, Kinshasa, Democratic Republic of the Congo; 13 Centre for Tropical Medicine and Global Health, Nuffield Department of Medicine, University of Oxford, Oxford, United Kingdom; 14 Department of Epidemiology of Microbial Diseases, New Haven, Connecticut, United States of America; 15 Yale Institute for Global Health, New Haven, Connecticut, United States of America; 16 Centre for International Health, Division of Health Sciences, University of Otago, Dunedin, New Zealand; 17 Institute for Health Metrics and Evaluation (IHME), University of Washington, Seattle, Washington, United States of America; 18 Department of Health Metrics Sciences, University of Washington, Seattle, Washington, United States of America; 19 Heidelberg Institute of Global Health, University of Heidelberg, Heidelberg, Germany; Huazhong University of Science and Technology Tongji Medical College, CHINA

## Abstract

**Background:**

With more than 1.2 million illnesses and 29,000 deaths in sub-Saharan Africa in 2017, typhoid fever continues to be a major public health problem. Effective control of the disease would benefit from an understanding of the subnational geospatial distribution of the disease incidence.

**Method:**

We collated records of the incidence rate of typhoid fever confirmed by culture of blood in Africa from 2000 to 2022. We estimated the typhoid incidence rate for sub-Saharan Africa on 20 km × 20 km grids by exploring the association with geospatial covariates representing access to improved water and sanitation, health conditions of the population, and environmental conditions.

**Results:**

We identified six published articles and one pre-print representing incidence rate estimates in 22 sites in 2000–2022. Estimated incidence rates showed geospatial variation at sub-national, national, and regional levels. The incidence rate was high in Western and Eastern African subregions followed by Southern and Middle African subregions. By age, the incidence rate was highest among 5–14 yo followed by 2–4 yo, > 14 yo, and 0–1 yo. When aggregated across all age classes and grids that comprise each country, predicted incidence rates ranged from 43.7 (95% confidence interval: 0.6 to 591.2) in Zimbabwe to 2,957.8 (95% CI: 20.8 to 4,245.2) in South Sudan per 100,000 person-years. Sub-national heterogeneity was evident with the coefficient of variation at the 20 km × 20 km grid-level ranging from 0.7 to 3.3 and was generally lower in high-incidence countries and widely varying in low-incidence countries.

**Conclusion:**

Our study provides estimates of 20 km × 20 km incidence rate of typhoid fever across sub-Saharan Africa based on data collected from 2000 through 2020. Increased understanding of the subnational geospatial variation of typhoid fever in Africa may inform more effective intervention programs by better targeting resources to heterogeneously disturbed disease risk.

## Author summary

Typhoid fever remains a significant health challenge in low- and middle-income nations, especially in sub-Saharan Africa. A comprehensive understanding of the disease’s geospatial distribution is pivotal for its control—a gap previous studies overlooked. Addressing this, we undertook a study to chart the incidence of typhoid fever throughout sub-Saharan Africa. Using data from 2000 to 2022, we developed high-resolution maps with a granularity of 20 km by 20 km, detailing the spatial distribution of typhoid incidence. Our findings reveal pronounced disparities in typhoid incidence across different geospatial tiers: from local communities to entire nations and regions. Particularly, Western and Eastern Africa registered the highest incidences, with children aged 5–14 years being the most vulnerable. Distinctively, countries such as South Sudan reported alarmingly high figures, whereas Zimbabwe had notably fewer cases. Such insights are indispensable for health policymakers at local, national, and global levels. Pinpointing the areas hardest hit by typhoid allows for a more strategic allocation of resources and interventions. Armed with this data, we’re better positioned to fight typhoid effectively and safeguard lives in sub-Saharan Africa.

## Introduction

Typhoid fever is a systemic infection caused by *Salmonella enterica* serovar Typhi (*S*. Typhi) that is transmitted via water or food contaminated by human feces [1]. Typhoid fever often causes mild symptoms such as fever or weakness but untreated may progress to potentially fatal complications such as intestinal perforation [2,3]. Typhoid fever continues to be a major public health problem in low- and middle-income countries (LMICs) [4–8]. While being the cornerstone for control of typhoid fever, improving water, sanitation, and hygiene (WASH) and food safety is generally a resource-intensive and long-term goal. Other control strategies such as vaccination, that may protect unvaccinated people as well as vaccine recipients [9], and antimicrobials, that may reduce morbidity and mortality among those with typhoid fever while also reducing fecal carriage and onward transmission [1], may facilitate more immediate impact and near-term control. Limited resources for health mean that global and national policy makers need to identify efficient ways to implement these control strategies, which demands detailed understanding of the distribution of disease.

Typhoid fever is estimated to have caused 14 million cases in 2010 and 11 million cases in 2017 globally [6]. Earlier studies reported somewhat varying estimates: 21 million cases worldwide in 2000 [7]; 27 million (interquartile range: 18–36 million) cases in 2010 [10]; 12 million (95% confidence interval: 10–15 million) or slightly higher in 2010 [4, 5]; and 17.8 million cases in 2015 but with much wider uncertainty intervals (95% credible interval: 6.9–48.4 million) [8].

Despite existing estimates, there are still knowledge gaps regarding the variation of typhoid fever incidence at the country or subnational levels. Understanding the subnational geospatial variation of the disease could lead to more efficient and effective intervention strategies by spatially targeting resources to areas at higher risk. Even for estimates that may agree at the global level, significant variation exists at the national and United Nations (UN) subregional levels [6]. Moreover, studies that estimate sub-national variation often rely on national-level covariates for modeling and prediction, potentially missing important local nuances [8]. In addition, existing studies do not account for observations during the recent typhoid surveillance in sub-Saharan Africa (SSA) and upating the estimates is necessary. Except for one prior study [8], the existing research has primarily examined the disease at the national level, neglecting sub-national variations. All estimates save two [5,6] rely on surveillance conducted in <20 sites before 2010, with only three sites from SSA including one from the 1980s.

We sought to address the limitations of existing studies and develop a more granular understanding of geospatial variation of typhoid fever incidence rates. In this study, we focused on SSA for which two multi-country surveillance studies, Typhoid Fever Surveillance in Africa Program (TSAP) conducted in 2012–2014, [11] and Severe Typhoid in Africa (SETA) conducted in 2016–2019 [12,13] provide recent data suggesting substatial burden of typhoid fever in the region. The issue of geospatial variation of typhoid fever is particualrly relevant to SSA because countries like the Democratic Republic of the Congo, Ghana, Malawi, and Zimbabwe are currently rolling out their typhoid vaccination programs [14–16]. We modeled age-stratified incidence rates by integrating data on the incidence rates and catchement area with recently synthesized geospatial covariates that represent factors that potentially influence the transmission of typhoid fever [17–20]. Using the predicted incidence rates on 20 km × 20 km grids of SSA, we characterized the geospatial variation of typhoid fever and potential implications.

## Methods

We modeled the incidence rate of typhoid fever on 20 km × 20 km grids by exploring the association of observed incidence rates with geospatial covariates representing factors that may influence the transmission of *S*. Typhi, such as access to improved water and sanitation, health conditions of the population, or environmental conditions. We chose the resolution of 20 km × 20 km because this resolution allows us to explore the subnational heterogeneity with reasonable computational cost. The expected number of 20 km × 20 km grids per country varies from about 28 (e.g., Gambia) to about 5,862 (e.g., Democratic Republic of the Congo). A previous study explored the subnational heterogeneity of the burden of cholera in sub-Saharan Africa at a similar resolution [21]. We used multivariate regression models, assuming the typhoid case count per 100,000 person-years followed either a Poisson or negative binomial distribution. The negative binomial distribution was considered to address overdispersion.

### Incidence rate data

**Fig 1:**
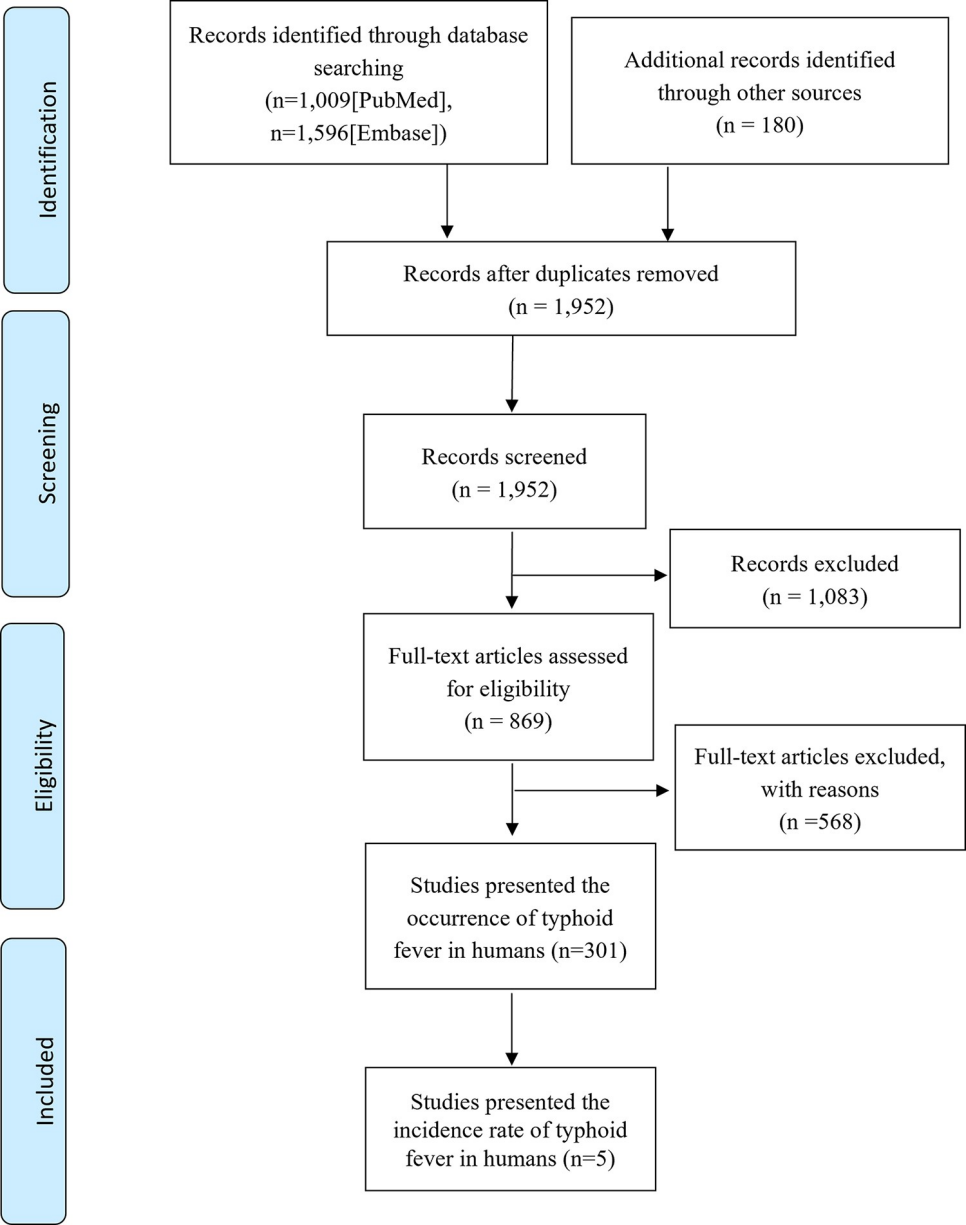
PRISMA flow diagram.

The data on incidence rates were extracted as a part of a broader project investigating the occurrence of typhoid fever, regardless of whether the instances were documented through case reports, outbreak investigations, or longitudinal surveillance. The literatures search procedure has been detailed elsewhere [22] and only studies that reported incidence rates from longitudinal surveillance were included in this study (**Fig 1**). In summary, we comprehensively searched through PubMed as well as preprint servers such as Social Science Research Network, bioRxiv, and medRxiv to identify relevant studies published between Jan 1, 2000 and Dec 31, 2022, using the search query "typhoid AND Africa.". Among 301 studies that reported occurrence of typhoid fever, five studies provided information on incidence rates of typhoid fever observed during longitudinal surveillance.

**Fig 2:**
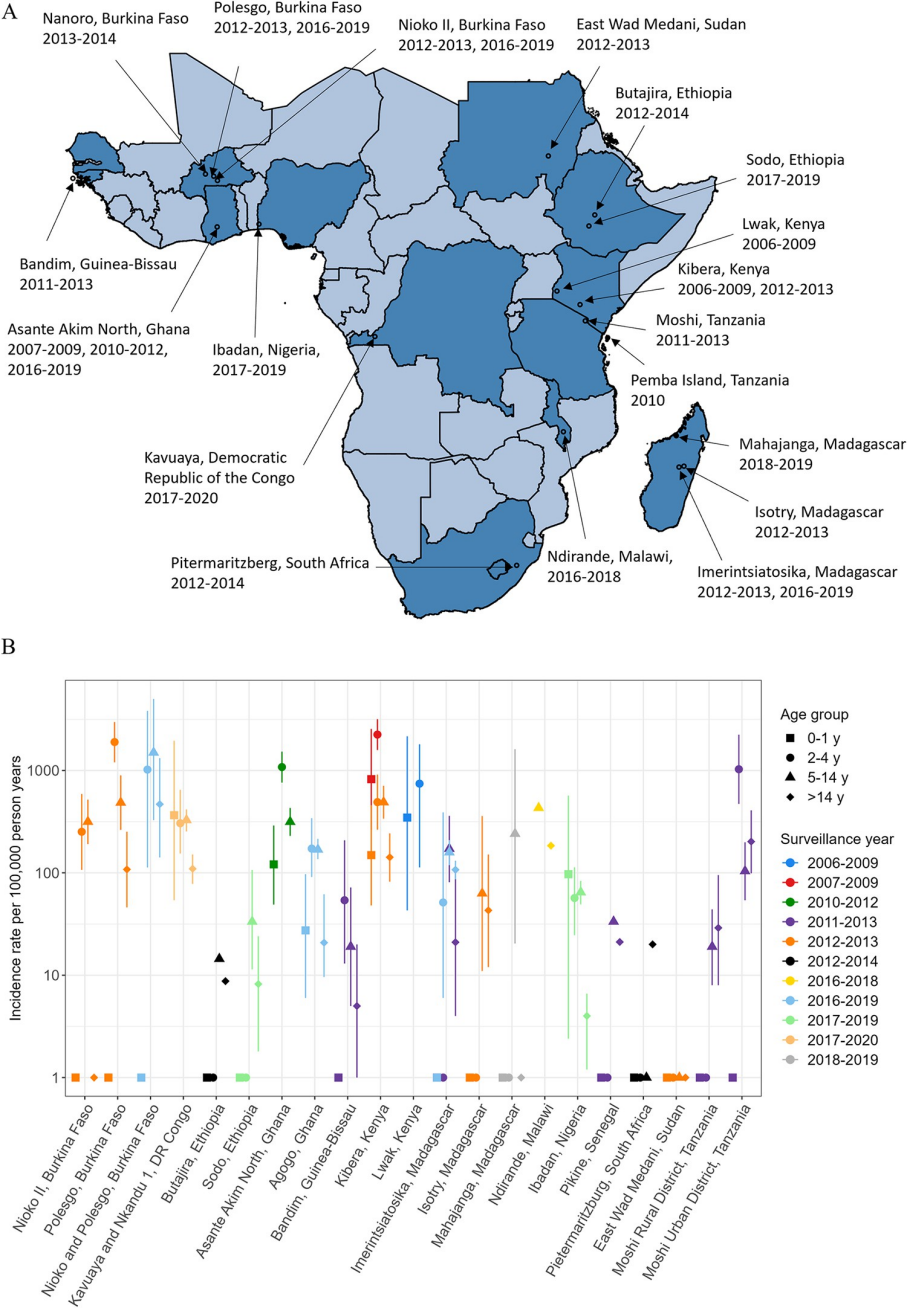
Observed typhoid incidence rates in sub-Saharan Africa, 2000–2020. (A) Year and location of the surveillance. (B) Incidence rates per 100,000 person-years that do not account for the sensitivity of blood culture tests. Points and bars indicate mean and 95% confidence or credible intervals. Points with no bars represent zero incidence rates in which interval estimates were not reported. Shapefiles specific to the African continent are available for download from GADM at: https://gadm.org/license.html.

Incidence rates for typhoid fever were available at 22 sites in 13 countries within SSA (**Fig 2** and **Table A** in S1 Information) [11,23–29]. Estimates were based on hospital-based passive surveillance with the exception of one study that involved active surveillance of households [28]. All studies used multipliers in their calculation of incidence rates to account for healthcare seeking behavior and the recruitment proportion; some studies used an additional multiplier to account for blood culture sensitivity [24,25,27]. We standardized the observed incidence rates across different studies by using the estimates that accounted for healthcare seeking behavior and recruitment proportion during the surveillance period. We later applied a uniform multiplier of 1/0.6 to modeled incidence rates to account for blood culture sensitivity (estimated to be ~60%) [4]. The incidence rates were broken down by age: 0–1, 2–4 yo, 5–14 yo, and >14 yo. Three studies [24,26,27] were not included in the analysis as they did not report typhoid incidence for the relevant age classes. One of these studies presented an aggregated incidence across all age classes [24], while the remaining two studies reported incidence among children under the age of 5 years [26] and children under the 15 years of age [27], respectively. One study used an age category of 5–17 years instead of 5–14 years [28]; we included this study in the model without further adjustment assuming incidence rate observed among those aged 5–17 years would be the same as the one observed in those 5–14 years.

To model incidence rates using geospatial covariates, we linked observed incidence rates from each surveillance site to the geospatial covariates that fall on the catchment area of the relevant healthcare facilities. Catchment areas were based on unpublished catchment area information collected during the surveillance (Section A and **Fig A** in S1 Information) [11] or the descriptions available in the studies [23–28].

### Geospatial covariates

Variables that may influence the water-borne or food-borne transmission of *S*. Typhi and cover the entire continent of SSA with at least 20 km × 20 km resolution were explored for their predictive capacity for the incidence rate of typhoid fever (**Table 1**). We explored access to safe drinking water and access to improved sanitation facilities [18,30–32] considering the water-borne or food-borne transmission of typhoid fever [1]. We included annual mean temperature [33–35], annual precipitation [34], elevation [36], and distance to water [37] because they may have the potential to influence the transmission of water-borne diseases and have been shown to be associated with an increased risk of typhoid fever. We also explored covariates that showed high predictive capacity in the previous modeling study [8], namely percent paved roads, percent of the population living in extreme poverty, prevalence of stunting, and prevalence of HIV [8]. Because the resolution of these covariates used in the previous modeling study [8] were low (i.e., national or first administrative unit level), we used high-resolution estimates that were recently synthesized: prevalence of stunting, wasting, and underweight under the age of five years [17] and the prevalence of HIV among adults aged 15–49 years [19]. We could not identify higher-resolution covariates for the two covariates—percent paved roads and people living in extreme poverty—and instead used a covariate indicating travel time to the nearest urban center [20] assuming all of these covariates partly capture infrastructure. We included human population density [38] as a covariate to reflect that the surveillance was conducted in sites with varying population density [39,40]. We created the population size for each 20 km × 20 km grids by aggregating the values from 1 km × 1 km grids using data from the WorldPop project [38].

**Table 1:** Characteristics of the candidate covariates included in the model.

Covariate	Description	Spatial resolution*	Temporal resolution* and duration	Ref.
Access to piped water	Percentage people with access to piped water (on or off premises)	5 km by 5 km	Yearly 2000–2017	[18]
Improved water	Percentage people with access to other improved drinking water facilities (protected wells and springs, bottled water, rainwater collection, bought water)	5 km by 5 km	Yearly 2000–2017	[18]
Access to surface water	Percentage people with access to surface water	5 km by 5 km	Yearly 2000–2017	[18]
Access to piped sanitation	Percentage people with access to septic or sewer sanitation	5 km by 5 km	Yearly 2000–2017	[18]
Access to improved sanitation	Percentage access to improved sanitation facilities (e.g., improved latrines, ventilated improved latrines, composting toilets)	5 km by 5 km	Yearly 2000–2017	[18]
Access to open defecation	Percentage people who practiced open defecation	5 km by 5 km	Yearly 2000–2017	[18]
Annual rainfall	Time mean flux of rain, snow and hail measured as the height of the equivalent liquid water in a square meter per day, which was then summed across the year	1 km by 1 km	Daily 2000–2017	[41]
Annual mean temperature	Temperature of air at a height of 2 metres above the Earth’s surface.	1 km by 1 km	Monthly 2000–2017	[41]
HIV prevalence	Prevalence of HIV among adults (aged 15–49 years)	5 km by 5 km	Yearly 2000–2017	[19]
Travel time to the nearest city	Travel time to the nearest urban center	1 km by 1 km	2015	[20]
Elevation	Elevation data from AWS Open Data Terrain Tiles	~30 m	Various data sources	[36]
Distance to water	The distance for each pixel to the nearest water cell (inland and sea)	~0.3 km by 0.3 km)	2015	[37]
Stunting prevalence	Proportion of children (0–59 months) with a height-for-age z-score that is more than two standard deviations below the World Health Organization’s median growth reference standards for a healthy population	5 km by 5 km	Yearly 2000–2017	[17]
Wasting	Proportion of children (0–59 months) with a weight-for-height that is more than two standard deviations below the World Health Organization’s median growth reference standards for a healthy population	5 km by 5 km	Yearly 2000–2017	[17]
Underweight	Proportion of children (0–59 months) with a weight-for-age z-score, that is more than two standard deviations below the World Health Organization’s median growth reference standards for a healthy population	5 km by 5 km	Yearly 2000–2017	[17]
Population density	The number of people in a grid cell	1 km by 1 km	Yearly 2000–2017	[38]

*Covariates were transformed to annual estimates on 20 km by 20 km grids before modeling

These geospatial covariates were based on the estimates from previous analyses. For instance, the access to water and sanitation facilities are the mean estimates from the geostatistical modeling analyses that were based on Demographic and Health Survey (DHS), Multiple Indicator Cluster Surveys, and other household surveys and censuses [18]. Similarly, prevalence of stunting, wasting, and underweight under the age of five years [17], and the prevalence of HIV among adults aged 15–49 years [19] were the mean estimates of previous geostatistical modeling analyses of the data from DHS and other sources. Other covariates such as temperature and precipitation came from satellite observations [41]. Elevation data came from ‘elevatr’ package [36], which uses Terrain Tiles Public Dataset [42]. These covariates were at finer resolutions (e.g., 5 km × 5 km or 1 km × 1 km) and created 20 km × 20 km grids by aggregating smaller grids and setting the value of the grid as the mean of the comprising smaller grids. Geospatial distribution of the covariates appears in **Figs B-Q** in S1 Information.

We matched the year and location of data collection between observed incidence rates and geospatial covariates. For covariates whose values were available for the period of 2000 through 2017, we assumed the values after 2017 were the same as in 2017. Elevation and distance to water covariates were assumed to be time-invariant. To determine the location for the observed incidence rates, we identified grid cells that were covered by the catchment area during the surveillance (using ‘extract’ function of raster package in R). A grid cell was defined to be covered by an area if its center was inside the area. The average value of the covariates across the grids was taken as the representative value when multiple grids were covered by the area.

### Predicting incidence rate

We employed a generalized linear modeling framework to explore the association between the outcome data and geospatial covariates. The outcome was quantified as case per 100,000 person-years for specific age groups (0–1 yo, 2–4 yo, 5–14 yo, and >14 yo) on 20 km × 20 km grids. Mathematical details about the models appear in Section B of S1 Information. For each model assuming a varying outcome distribution (Poisson or negative binomial), we conducted the following variable selection process. We removed covariates whose variance inflation factors (VIFs) were over ten, or that were highly correlated with other variables (Pearson’s *r* > 0.7) while having low correlation with the outcome (Pearson’s *r* < 0.1) to reduce multicollinearity [43]. We performed a forward stepwise variable selection method based on Akaike Information Criterion (AIC) [44] because AIC criteria can be straightforwardly applied to generalized models, including generalized linear models, non-linear models, and non-normal distributed data. A covariate was included in the model if its introduction led to a decrease in the AIC. Conversely, the covariate was not included in the model if its introduction didn’t lead to a decrease in AIC. The covariate with the highest positive or negative correlation with the outcome was tested first, followed by the covariate with the next highest correlation with the outcome, and so on. We further selected covariates based on their *p*-values, their impact on root mean-square error (RMSE) in the leave-one-out (LOO) cross-validation [45] (Section C in S1 Information), and the age-distribution of the predicted incidence rates. We retained variables if their *p*-values were lower than 0.05, their addition reduced the RMSE in the LOO cross-validation, and they produced incidence rate that resembled the data in age distribution (i.e., incidence rates higher in 2–14 yo than in 0–1 yo and >14 yo). We chose the Poisson model for 0–1 yo and the negative binomial models for the other age classes. A list of covariates included in the final model and their associated parameter estimates appear in **Table B** in S1 Information.

To calculate the predicted number of cases at the grid level, we multiplied the predicted incidence rates with the population size for each 20 km × 20 km grid. Since the WorldPop data lacked age-specific population sizes, we estimated the age-specific population sizes by multiplying the population size at the grid level with the overall proportions of each age class at the country level, sourced from information in the UN World Population Prospectus [46]. To determine the predicted number of cases at the country level, we aggregated the grid-level cases across all the grids within the country of interest. Subsequently, we computed the country-level incidence rate by dividing the number of cases by the population size at the country level. To compare the predicted number of cases with estimates of previous studies, we assumed blood culture sensitivity at 60% as in the previous study [4] and used population size from 2017 [6,8]. We set the upper bound of the predicted incidence rates to 10,000 per 100,000 person-years if the predicted incidence rates were higher than 10,000 per 100,000 person-years.

### Data sharing

All data and R code required to replicate the figures and tables presented in this paper are accessible in the GitHub repository [47]. Shapefiles specific to the African continent are available for download from GADM at: https://gadm.org/license.html. Information about the sources for all other publicly available data used in this study is documented in the respective sections where these data are referenced.

## Results

### Model and predictor selection

The negative binomial regression model yielded the best predictive performance for age groups 2–4 yo, 5–14 yo, and >14 yo, while the Poisson model demonstrated superior performance for the 0–1 yo group (refer to Table C in S1 Information for RMSE values). These models with the best performance were then employed to predict the incidence rates of typhoid for the corresponding age group on 20 km × 20 km grids. Across all models, the covariates measuring the distance to water bodies and the proportion of people using surface water were included, and both demonstrated a positive association with the typhoid incidence rate, but to a varying extent (refer to **Table 2**). However, different covariates were additionally selected and therefore the final set of covariates varied by age group. For the 0–1 yo age group, population density and open defecation exhibited a positive association, whereas elevation, access to piped sanitation, and wasting showed a negative association with the incidence rate. For the 2–4 yo age group, only population density was additionally included in the model, and it had a positive association. For the 5–14 yo age group, only the proportion of population who have access to piped water was additionally included in the model, and it had a positive association.

**Table 2:** The model with the final set of covariates for each group. Blank cells indicate the variables that were excluded during the variable selection process.

Variable	0–1 yo			2–4 yo			5–14 yo			>14 yo		
	Est.	SE	*p*-value	Est.	SE	*p*-value	Est.	SE	*p*-value	Est.	SE	*p*-value
Improved water				0.034	0.023	0.149						
Improved sanitation												
Annual rainfall										0.691	0.238	0.004
Annual mean temperature												
Stunting prevalence										11.935	4.332	0.006
HIV prevalence												
Travel time to the nearest city												
Elevation	-3.848	0.860	< 0.001									
Distance to water	0.082	0.017	< 0.001	0.088	0.042	0.038	0.064	0.022	0.004	0.101	0.028	0.000
Access to piped water							0.029	0.008	0.000	0.043	0.013	0.001
Access to piped sanitation	-0.112	0.024	< 0.001							0.028	0.013	0.029
Access to surface water	0.079	0.015	< 0.001	0.080	0.039	0.040	0.036	0.018	0.049	0.104	0.024	0.000
Access to open defecation	0.068	0.014	< 0.001									
Wasting	-70.458	11.988	< 0.001									
Underweight												
Population density	1.312	0.210	0.000	0.398	0.259	0.125						

Est. = estimates; SE = standard error

### Comparison between observed and predicted incidence rates

Overall, 50 incidence rate estimates were available for 0–1 yo, 2–4 yo, 5–14 yo, and >14 yo age classes for 22 sites in 13 countries in sub-Saharan Africa (**Table A** in S1 Information). All studies reported incidence rates for all age classes except for one study [26] reporting incidence rates for those who were 4 years old or younger. Surveillance sites included both urban and rural sites and provided incidence rates observed over the period from 2006 through 2019. The surveillance duration for each site ranged from 13 months (East Wad Medani, Sudan for July 2012—July 2013) to >5 years (Asante Akim North, Ghana for March 2010—May 2012 and May 2016—May 2019).

**Fig 3:**
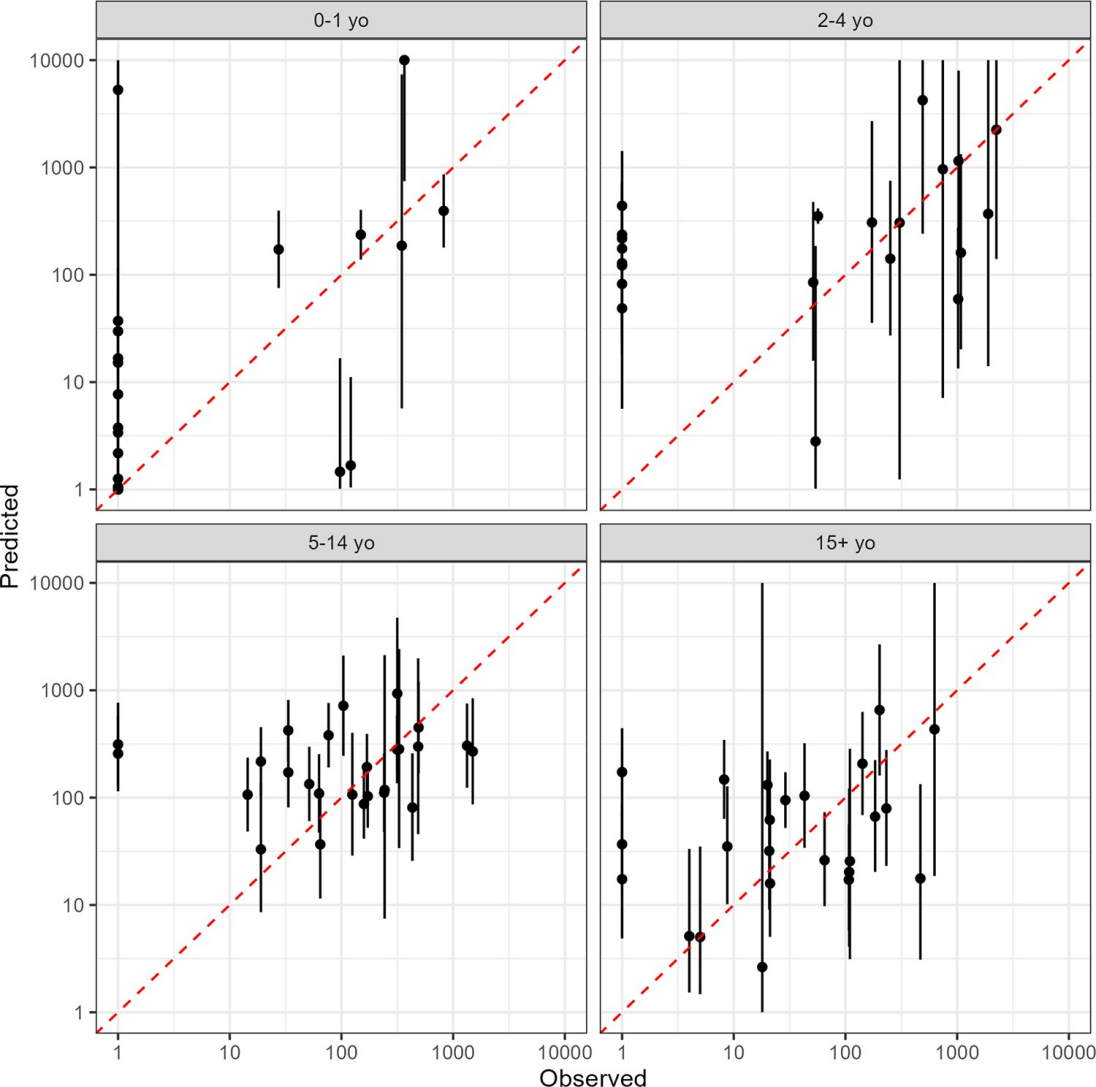
Leave-one-out cross-validation for age-stratified typhoid incidence rate estimates. Predicted against observed incidence rates per 100,000 person-years on log-log scale. Dots and bars represent the mean and 95% confidence intervals of predicted values.

A substantial fraction of the observed incidence rates was predicted by the model in the LOO cross-validation (**Fig 3**). For the 0–1 yo, 2–4 yo, 5–14 yo, and >14 yo age groups, 13.6%, 50.0%, 53.8%, 62.5% of the observations were within the 95% confidence interval of the predictions, respectively.

### Predicted incidence rate

**Fig 4:**
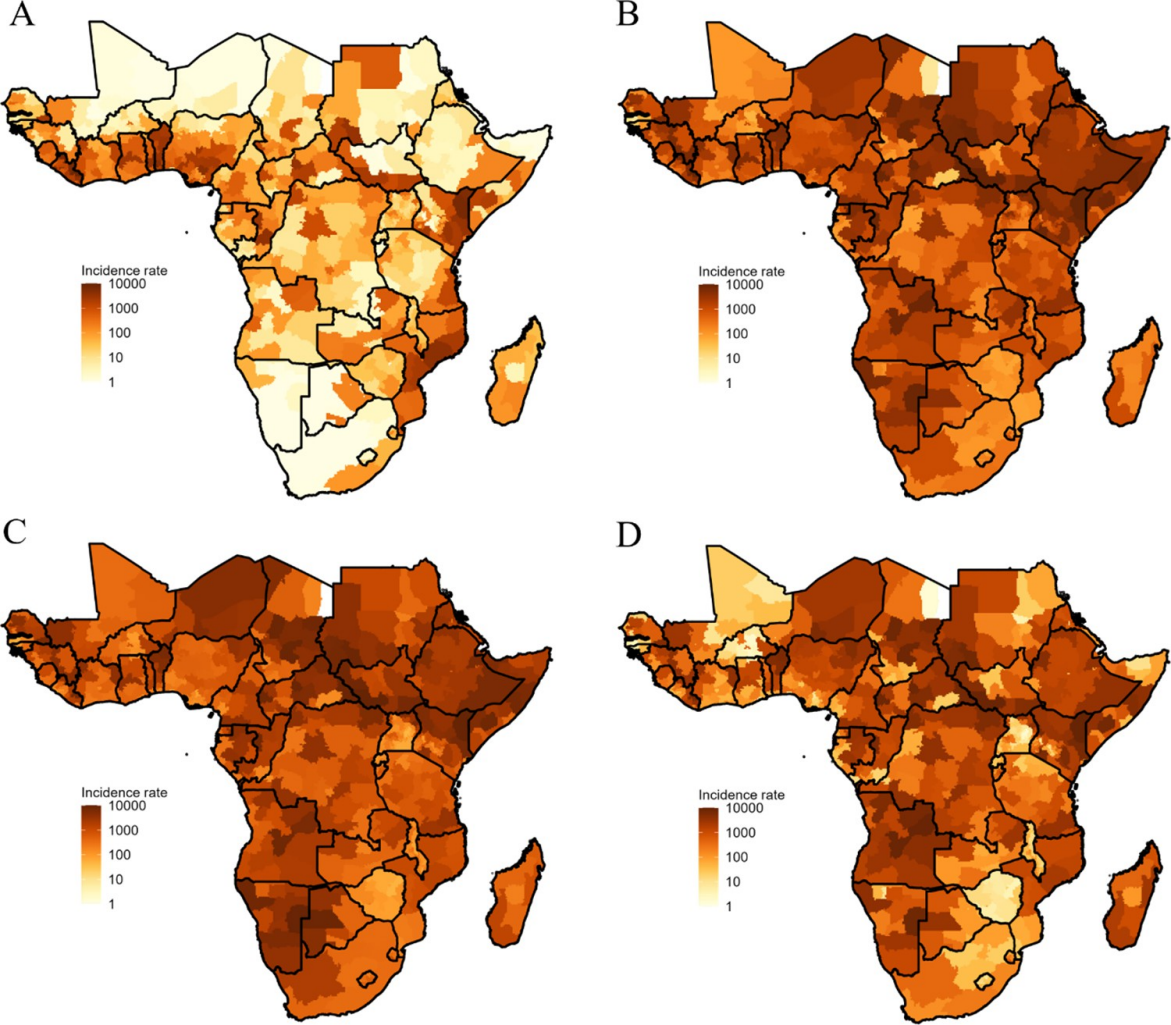
Predicted incidence rates per 100,000 person-years for 0–1 yo (A), 2–4 yo (B), 5–14 yo (C), and >14 yo (D), summarized at subnational levels for 2017. Shapefiles specific to the African continent are available for download from GADM at: https://gadm.org/license.html.

Predicted incidence rates varied at the grid, subnational, country, and subregional levels. For instance, variations at the subnational level were evident in all age classes (**Fig 4A–4D**). Overall, 2–4 yo were predicted to have the highest incidence rates (417.5 [95% CI: 10.7 to 7291.5] per 100,000 person-years) followed by 5–14 yo (308.0 [95% CI: 33.7 to 6590.6]), and > 14 yo (46.2 [95% CI: 1.0 to 5661.3]); 0–1 yo were predicted to have the lowest incidence rates (21.0 [95% CI: 0.0 to 2840.7]) (**Fig R** in S1 Information).

**Fig 5:**
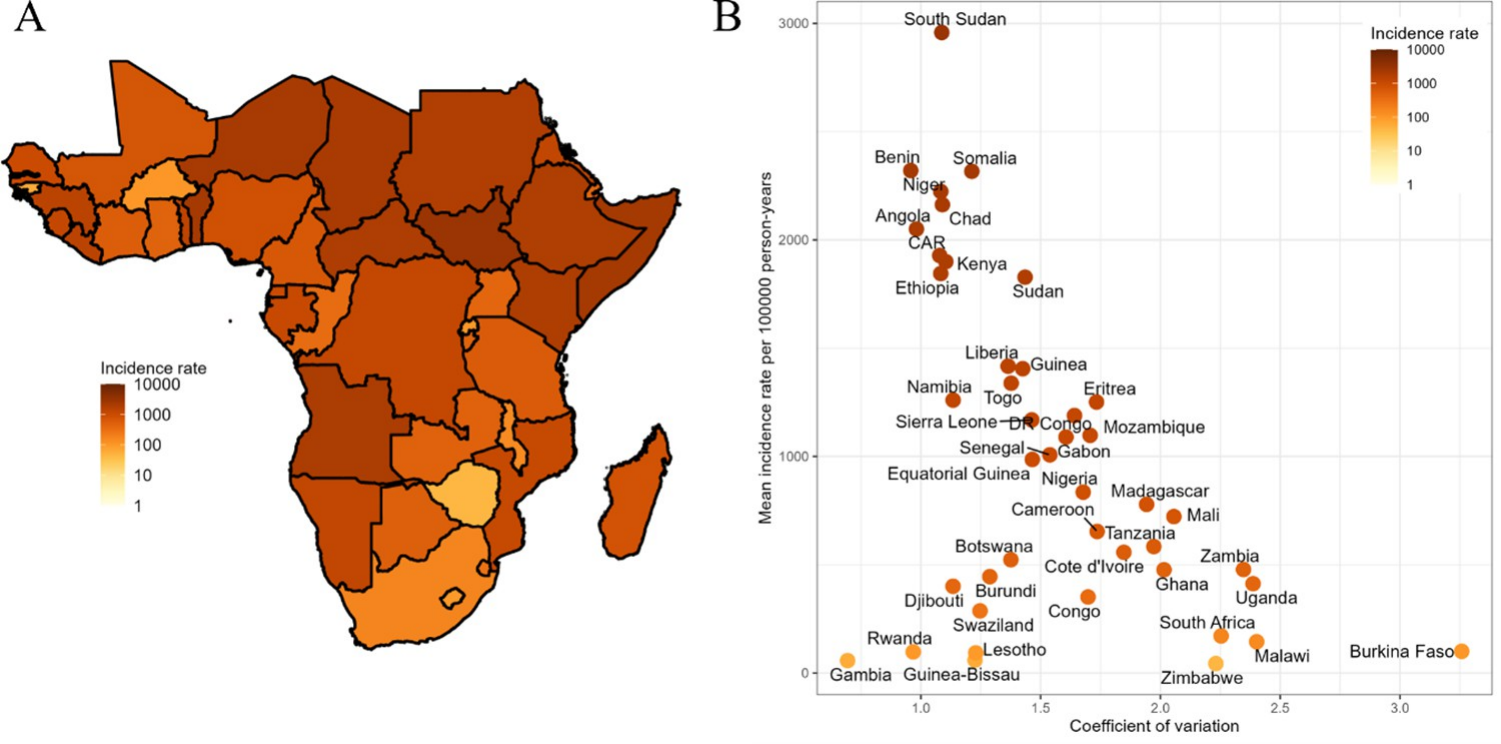
Country-level typhoid incidence rates per 100,000 person-years (A) and the coefficient of variation (CV) of 20 km × 20 km grid-level incidence rates (B). Mean country incidence rates varied from 43.7 to 2,957.8 and CV varies from 0.7 to 3.3. Shapefiles specific to the African continent are available for download from GADM at: https://gadm.org/license.html.

Country-level variation of the predicted incidence is also evident in all age groups (**Fig S** and **Table D** in S1 Information). When aggregated across all age classes and grids that comprise each country, predicted incidence rates ranged from 43.7 (95% CI: 0.6 to 591.2) in Zimbabwe to 2,957.8 (95% CI: 20.8 to 4,245.2) in South Sudan per 100,000 person-years (**Fig 5**, **Table D** in S1 Information). At the UN subregional level [48], Middle Africa had the highest incidence rate (1,017.1 [95% CI: 32.0 to 1,275.6] per 100,000 person-years), followed by Eastern Africa, Western Africa, and Southern Africa (**Table E** in S1 Information).

The expected annual number of cases in the continent adjusted for the 2017 population was 1,883,572 (95% CI: 127,474 to 6,535,604). By UN subregion, the number of typhoid fever cases was highest in Eastern Africa (4,782,220 [95% CI: 277,999 to 11,897,898] cases) followed by Western Africa (3,544,420 [95% CI: 1,904 to 9,874,415]), Middle Africa (2,515,459 [95% CI: 98,917 to 5,479,315]), and Southern Africa (145,130 [95% CI: 1,904 to 749,018]) (**Tables F and G** in S1 Information). Countries with high typhoid incidence (e.g., South Sudan) were associated with the lower coefficient of variation (CV) of 20 km × 20 km grid-level incidence rates whereas low-incidence countries were associated with a wide range of CVs from higher (e.g., Burkina Faso) and lower (e.g., Gambia) **(Fig 5)**.

## Discussion

We estimated the age-specific incidence rates of typhoid fever for SSA on 20 km × 20 km grids using typhoid incidence rate data over the period of 2000 through 2022 including recently completed surveillance [13] and high-resolution geospatial covariates matched to the catchment area for the surveillance sites. Predicted incidence rates showed substantial geospatial variation at all levels: subnational, national and Africa subregional levels. To the best of our knowledge, this study represents the first exploration of the high-resolution distribution of typhoid incidence rates across the African continent, using the most current available data. While extracting actionable insights directly from the study results may pose challenges, achieving a more comprehensive understanding of the geospatial distribution of typhoid burden by age could lead to more effective and efficient targeted intervention programs [49,50].

While our model primarily focuses on predicting incidence rates per 100,000 people rather than providing explanations for the association between covariates and incidence rates, the selected covariates in the model appear to offer reasonable explanations. For instance, using surface water (all age groups), population density (0–1 yo and 2–4 yo age groups), and open defecation (0–1 yo) appear to show a positive association with typhoid incidence per capita. Conversely, covariates like access to piped sanitation and elevation indicate a negative association with the incidence rate, whereas a negative association between the prevalence of wasting and the incidence rate is not intuitively clear.

Existing estimates of the typhoid fever burden generally align on a global scale but exhibit considerable variation at the UN subregional level [6]. Our UN subregional-level estimates, overall, tend to surpass those of Mogasale*et al*.[4] and GBD estimates [6] while closely resembling the estimates presented by Antillón *et al*.[8]. Notably, there’s a substantial disparity for Eastern Africa, where our estimates are approximately twice as high as those reported by Antillón *et al*.[8]. (**Tables E** and **G** in S1 Information**).** However, due to the limited number of observations, both our estimates and those of by Antillón *et al*.[8] display significant uncertainty ranges, which considerably overlap and encompass the estimates of Mogasale *et al*. [4] whereas GBD estimates [6] persistently remain lower without overlapping with other estimates.

Intrepreting agreement or disagreement among estimates should be done with care, however, and needs to account for the context of the models and the data used in each study. For instance, estimates by Mogasale *et al*.[4] and Antillón *et al*.[8] did not account for the data from TSAP and SETA [11, 12], which are a major source of data for incidence rate modeling in our study. Also, the methods used in the studies by Mogasale *et al*.[4] and Kim *et al*.[5] do not use any geospatial covariates to model observed incidence rates except for national-level estimates for the access to improved water source.

Incidence rate estimates from this study were characterized by large uncertainties. One of the main reasons for the wide confidence intervals is the small sample size. While we included all the datasets we found in the peer-reviewed literature including one pre-print as of January 2023, the number of observations was fewer than 30 for all age groups. The 95% confidence intervals of estimates from this study overlapped substantially with those of Antillón *et al*.[8] and encapsulate estimates of other studies (**Table I** and **Fig T** in S1 Information); however, the lower bounds of the confidence intervals tended to be lower than those of other studies with many of them close to zero, which might reflect the fact that the data set we used included zeros in 20% of the data.

Our study has limitations. First, while population-based surveillance provides reliable inputs for the model to predict the incidence of typhoid fever, these data were available from only 22 sites in 13 countries [11,13,23–28], which makes it challenging to infer incidence for all of SSA. While research collecting additional data and developing ways to use other information such as serosurveys [51] should continue, use of the most up-to-date incidence rate data and high-resolution geospatial covariates enable us to produce updated incidence rates at the subnational level. Second, like the estimates of previous studies for typhoid and other diseases [4–8,10,52], our estimates are predictions of annual incidence rates that are based on static and retrospective analyses. While these estimates can provide clues to the current incidence of disease, the burden of typhoid fever is seasonally and secularly variable, and effective policy making would require real-time data synthesis. Third, we set an upper limit for the incidence rates as 10,000 per 100,000 person-years, which is around 4 times higher than the highest of the observed incidence rates, while some of the model estimates at 20 km × 20 km grids go beyond the limit. This may introduce a bias that incidence rate estimates are lower for some areas. For 0–1 yo, 2–4 yo, 5–14 yo, and >14 yo, 0.2%, 5.6%, 6.2%, 5.1% of the total 60,535 valid grids had estimated values over 10,000 per 100,000 person-years. Fourth, while we used granular geospatial covariates to model the incidence rates, we had to rely on the nationally averaged age distribution to produce the age-specific predicted number of cases. Therefore, age-specific predicted number of cases may either overestimate or underestimate the true number of cases. Fifth, we modeled incidence rates of typhoid fever separately for each age group and this led to different sets of covariates for different age groups. Associations with the covariate in the models should not be interpreted as causal and many vary by age to the extent that certain covariates are not significant predictors of incidence in all age groups. Sixth, the predictive power of our model is limited for 0–1 yo, potentially because a large fraction of observations are zeroes. This warrants additional data and updates.

## Conclusion

Our research offers the most detailed prediction to date of the typhoid fever incidence rate in Sub-Saharan Africa (SSA), utilizing the latest data on incidence rates and geospatial variables at a resolution of 20 km × 20 km. This study enhances understanding of the geographical variations in typhoid fever and provides valuable data that could inform targeted intervention strategies for typhoid control in the region.

## Supporting information

S1 Information Table A. Longitudinal surveillance studies of typhoid fever incidence in Africa. Incidence rate data used for modeling come from four published articles and one preprint surveillance study that reported incidence rates measures in sub-Saharan Africa since 2000. **Table B**. Estimated coefficients of covariates in the proposed model, sub-Saharan Africa, 2017. Greyed cells indicate variables that were removed before modeling to reduce multicollinearity. Cells with the blue background indicate variables that were removed because the p-values were larger than or near to 0.05 and excluding them reduced the LOO cross-validation RMSE. **Section A.** Catchment area for the incidence rates **Fig A.** Catchment area represented as 20 km × 20 km grids. Shapefiles specific to the African continent are available for download from GADM at: https://gadm.org/license.html. **Fig B**. Percentage access to improved sanitation facilities based on Deshpande *et al*. [18]. The improved sanitation includes sewer or septic tanks and other improved sanitation facilities (improved latrines, ventilated improved latrines, composting toilets). The dataset is available at the IHME: https://cloud.ihme.washington.edu/s/bkH2X2tFQMejMxy. **Fig C**. Percentage access to sewer or septic sanitation facilities based on Deshpande *et al*. [18]. The dataset is available at the IHME: ttps://cloud.ihme.washington.edu/s/bkH2X2tFQMejMxy. **Fig D**. Percentage open defecation based on Deshpande *et al*. [18]. The dataset is available at the IHME: https://cloud.ihme.washington.edu/s/bkH2X2tFQMejMxy. **Fig E**. Access to improved drinking water based on Deshpande *et al*. [18]. The improved water indicates access to piped water according to the JMP definition and includes piped (piped on or off premises) and other improved (protected wells and springs, bottled water, rainwater collection, bought water) water. The dataset is available at the IHME: https://cloud.ihme.washington.edu/s/bkH2X2tFQMejMxy. **Fig F**. Access to piped drinking water based on Deshpande *et al*. [18]. The dataset is available at the IHME: https://cloud.ihme.washington.edu/s/bkH2X2tFQMejMxy. **Fig G**. Use of surface water based on Deshpande *et al*. [18]. The dataset is available at the IHME: https://cloud.ihme.washington.edu/s/bkH2X2tFQMejMxy. **Fig H**. Precipitation. The values indicate annual precipitation summed across daily precipitation data Climate Hazards group Infrared Precipitation with Stations (CHIRPS) data set [41]. The dataset is available at https://cds.climate.copernicus.eu/cdsapp#!/dataset/insitu-gridded-observations-global-and-regional?tab=form. Shapefiles specific to the African continent are available for download from GADM at: https://gadm.org/license.html. **Fig I**. Annual mean temperature for 2017. Monthly mean temperatures [41] were averaged. The dataset is available at https://cds.climate.copernicus.eu/cdsapp#!/dataset/insitu-gridded-observations-global-and-regional?tab=form. Shapefiles specific to the African continent are available for download from GADM at: https://gadm.org/license.html. **Fig J**. Prevalence of stunting among children under the age of 5 in Africa for 2017 based on the study by Kinyoki *et al*. [17]. The dataset is available at https://cloud.ihme.washington.edu/index.php/s/Q5CGeazb4iNsDQA. Shapefiles specific to the African continent are available for download from GADM at: https://gadm.org/license.html. **Fig K**. Prevalence of wasting among children under the age of 5 in Africa for 2017 based on the study by Kinyoki *et al*. [17]. The dataset is available at https://cloud.ihme.washington.edu/index.php/s/Q5CGeazb4iNsDQA. Shapefiles specific to the African continent are available for download from GADM at: https://gadm.org/license.html. **Fig L**. Prevalence of underweight among children under the age of 5 in Africa for 2017 based on the study by Kinyoki *et al*. [17]. The dataset is available at https://cloud.ihme.washington.edu/index.php/s/Q5CGeazb4iNsDQA. Shapefiles specific to the African continent are available for download from GADM at: https://gadm.org/license.html. **Fig M.** Prevalence of HIV infection among adults (15–49 years old) in sub-Saharan Africa for 2017 based on the study by Dwyer-Lindgren *et al*.[19]. The dataset is available at https://ghdx.healthdata.org/record/ihme-data/africa-hiv-prevalence-geospatial-estimates-2000-2017. Shapefiles specific to the African continent are available for download from GADM at: https://gadm.org/license.html. **Fig N**. Travel time to cities (in minutes) in Africa for 2017 based on the study by Weiss *et al*. [20]. The dataset is available at https://data.malariaatlas.org/maps. Shapefiles specific to the African continent are available for download from GADM at: https://gadm.org/license.html. **Fig O**. Distance to water [37]. The value indicates the distance for each pixel to the nearest water cell (inland and sea) at 20 km × 20 km resolution. For the water cell the distance to water is 0 km. The dataset is available at https://data.ceda.ac.uk/neodc/globolakes/data/v1/limnology. Shapefiles specific to the African continent are available for download from GADM at: https://gadm.org/license.html. **Fig P**. Elevation [36]. Elevation data come from estimates by Mapzen that combine several digital elevation model (DEM) such as the Shuttle Radar Topography Mission (SRTM), the USGS National Elevation Dataset (NED), Global DEM (GDEM), and others. R package ‘elevatr’ serves as an API that enables an access to the elevation estimates by Mapzen hosted at Amazon Web Services Terrain Tiles. Shapefiles specific to the African continent are available for download from GADM at: https://gadm.org/license.html. **Fig Q**. Population count per pixel based on the WorldPop [38]. Mosaiced 1km resolution global dataset were aggregated to create 20 km resolution dataset. The dataset is available at https://hub.worldpop.org/geodata/listing?id=64. Shapefiles specific to the African continent are available for download from GADM at: https://gadm.org/license.html. **Section B**. Multivariate regression for Poisson and negative binomial models. **Section C.** Model validation. **Table C.** The root mean squared error (RMSE) values of the proposed model (linear regression model) with the competing models (Poisson and Negative Binomial regression models). **Fig R.** Distribution of predicted incidence rates per 100,000 person years by age (A) and incidence rate ratio (B) with children aged 5–14 years as a reference group. Incidence rates were summarized at subnational levels. Red dots indicate observed incidence rates. **Fig S.** Predicted incidence rates per 100,000 person-years for 0–1 yo (A), 2–4 yo (B), 5–14 yo (C), and >14 yo (D) summarized at country level. Bold and thin lines inside Africa represent country borders and first-level administrative divisions, respectively. Shapefiles specific to the African continent are available for download from GADM at: https://gadm.org/license.html. **Table D.** Estimated incidence rates per 100,000 person years by country, sub-Saharan Africa, 2017. **Table E.** Estimated incidence rate of typhoid fever per 100,000 persons per year by Africa subregion, 2017. **Table F.** Estimated number of cases by country and age group, sub-Saharan Africa, 2017. **Table G.** Estimated number of cases by Africa subregion, 2017 (unit = thousands). **Table H.** Comparison of incidence rates per 100, 000 person-years by country. **Fig T**. Incidence rate estimates per 100,000 person-years by country. (A) shows existing incidence rate estimates at the country level. The upper bounds of some estimates by Antillón *et al*. and in the current study go over 4,000. (B) highlights the differences between the estimates by Antillón *et al*. and by this study that take a similar approach of using grid-level geospatial covariates.
(DOCX)
