# Supplementary material for: Mapping the incidence rate of typhoid fever in sub-Saharan Africa
Source: PLoS Negl Trop Dis. 2024 Feb 26;18(2):e0011902. doi: 10.1371/journal.pntd.0011902 (PMC10965079; doi:10.1371/journal.pntd.0011902)
Supplement: S1 Information — Incidence rate data used for modeling come from four published articles and one preprint surveillance study that reported incidence rates measures in sub-Saharan Africa since 2000. Table B. Estimated coefficients of covariates in the proposed model, sub-Saharan Africa, 2017. Greyed cells indicate variables that were removed before modeling to reduce multicollinearity. Cells with the blue background indicate variables that were removed because the p-values were larger than or near to 0.05 and excluding them reduced the LOO cross-validation RMSE. Section A. Catchment area for the incidence rates Fig A. Catchment area represented as 20 km × 20 km grids. Shapefiles specific to the African continent are available for download from GADM at: https://gadm.org/license.html. Fig B. Percentage access to improved sanitation facilities based on Deshpande et al. [18]. The improved sanitation includes sewer or septic tanks and other improved sanitation facilities (improved latrines, ventilated improved latrines, composting toilets). The dataset is available at the IHME: https://cloud.ihme.washington.edu/s/bkH2X2tFQMejMxy. Fig C. Percentage access to sewer or septic sanitation facilities based on Deshpande et al. [18]. The dataset is available at the IHME: ttps://cloud.ihme.washington.edu/s/bkH2X2tFQMejMxy. Fig D. Percentage open defecation based on Deshpande et al. [18]. The dataset is available at the IHME: https://cloud.ihme.washington.edu/s/bkH2X2tFQMejMxy. Fig E. Access to improved drinking water based on Deshpande et al. [18]. The improved water indicates access to piped water according to the JMP definition and includes piped (piped on or off premises) and other improved (protected wells and springs, bottled water, rainwater collection, bought water) water. The dataset is available at the IHME: https://cloud.ihme.washington.edu/s/bkH2X2tFQMejMxy. Fig F. Access to piped drinking water based on Deshpande et al. [18]. The dataset is available at the IHME: https://cloud.ihme.was [file pntd.0011902.s001.docx]

# S1 information

# Title

Mapping the incidence rate of typhoid fever in sub-Saharan Africa

# Authors

Jong-Hoon Kim^1#^, Jungsoon Choi^2^, Chaelin Kim^1^, Gi Deok Pak^1^, Prerana Parajulee^1^, Andrea Haselbeck^1^, Se-Eun Park^1,3^, Vittal Mogasale^4^, Hyon Jin Jeon1^5^, Annie J Browne^6^, Ellis Owusu-Dabo^8^, Raphaël Rakotozandrindrainy^9^, Abdramane Soura Bassiahi^10^, Mekonnen Teferi^11^, Octavie Lunguya-Metila^12,13^, Christiane Dolecek^14^, Virginia E. Pitzer^15,16^, John A. Crump^17^, Simon I. Hay^18,19^, Florian Marks^1,5,9,20^

**Affiliations**

^1^International Vaccine Institute, Seoul, Republic of Korea.

^2^Department of Mathematics, Hanyang University, Seoul, Republic of Korea

^3^Graduate School of Public Health, Yonsei University, Seoul, Republic of Korea

^4^International Vaccine Institute, Seoul, Republic of Korea (Current affiliation: Department of Health Systems Governance and Financing, World Health Organization, Geneva, Switzerland)

^5^Cambridge Institute of Therapeutic Immunology and Infectious Disease, University of Cambridge School of Clinical Medicine, Cambridge Biomedical Campus, Cambridge, UK, CB2 0AW

^6^Malaria Atlas Project, Telethon Kids Institute, Perth, Australia.

^8^School of Public Health, Kwame Nkrumah University of Science and Technology, Laing Building Complex J.W. Acheampong CI, Kumasi, Ghana

^9^Madagascar Institute for Vaccine Research, University of Antananarivo, 3HM2+QH7, Antananarivo, Madagascar

^10^Institut Supérieur des Sciences de la Population, 03 BP 7118, Blvd Charles De Gaulle, Ouagadougou, Burkina Faso

^11^Armauer Hansen Research Institute, ALERT Compound Zenebework, Jimma Road, Addis Ababa 1005, Ethiopia

^12^Department of Microbiology, Institut National de Recherche Biomédicale, M8R2+4WX, Kinshasa, Democratic Republic of Congo

^13^Department of Medical Biology, Microbiology Service, University Teaching Hospital, M8R4+CF3, Ave De L'hopital, Kinshasa, Democratic Republic of the Congo

^14^Centre for Tropical Medicine and Global Health, Nuffield Department of Medicine, University of Oxford, Oxford, UK

^15^Department of Epidemiology of Microbial Diseases, New Haven, Connecticut, US

^16^Yale Institute for Global Health, New Haven, Connecticut, US

^17^Centre for International Health, Division of Health Sciences, University of Otago, Dunedin, New Zealand

^18^Institute for Health Metrics and Evaluation (IHME), University of Washington, Seattle, Washington, US

^19^Department of Health Metrics Sciences, University of Washington, Seattle, Washington, US

^20^Heidelberg Institute of Global Health, University of Heidelberg, Heidelberg, Germany

**Table A** in S1 Information. Longitudinal surveillance studies of typhoid fever incidence in Africa. Incidence rate data used for modeling come from four published articles and one preprint surveillance study that reported incidence rates measures in sub-Saharan Africa since 2000.

| Ref | Country | Site | Year | Age group | Incidence rate per 100,000 person years  (95% confidence interval) |
| --- | --- | --- | --- | --- | --- |
| Breiman [1] (2012) | Kenya | Kibera | 2007 - 2009 | 0-1 y | 821.5 (265 - 2547) |
|  |  |  |  | 2-4 y | 2242.6 (1586 - 3171) |
|  |  |  |  | 5-9 y | 1788 (1348 - 2373) |
|  |  |  |  | 10-17 y | 869.9 (583 - 1298) |
|  |  |  |  | >17 y | 231.3 (160 - 335) |
|  |  | Lwak | 2006 - 2009 | 0-1 y | 345.7 (43 - 2158) |
|  |  |  |  | 2-4 y | 742.6 (113 - 1804) |
|  |  |  |  | 5-9 y | 215.5 (56 - 903) |
|  |  |  |  | 10-17 y | 260.4 (108 - 767) |
|  |  |  |  | >17 y | 231.3 (160 - 335) |
| Marks [2] (2017)^*^ | Burkina Faso | Nioko II | 2012 - 2013 | 0-1 y | 0 |
|  |  |  |  | 2-4 y | 251 (107 - 590) |
|  |  |  |  | 5-14 y | 315 (191 - 519) |
|  |  |  |  | >14 y | 0 |
|  |  | Polesgo | 2012 - 2013 | 0-1 y | 0 |
|  |  |  |  | 2-4 y | 1895.2 (1202 - 2972) |
|  |  |  |  | 5-14 y | 485 (263 - 896) |
|  |  |  |  | >14 y | 107 (46 - 252) |
|  | Ethiopia | Butajira | 2012 - 2014 | 0-1 y | 0 |
|  |  |  |  | 2-4 y | 0 |
|  |  |  |  | 5-14 y | NA |
|  |  |  |  | >14 y | NA |
|  | Ghana | Asante Akim North | 2010 - 2012 | 0-1 y | 120 (49 - 290) |
|  |  |  |  | 2-4 y | 1079 (762 - 1528) |
|  |  |  |  | 5-14 y | 314 (230 - 430) |
|  | Guinea-Bissau | Bandim | 2011 - 2013 | 0-1 y | 0 |
|  |  |  |  | 2-4 y | 53 (13 - 208) |
|  |  |  |  | 5-14 y | 18 (5 - 72) |
|  |  |  |  | >14 y | 4 (1 - 20) |
|  | Kenya | Kibera | 2012 - 2013 | 0-1 y | 148 (48 - 458) |
|  |  |  |  | 2-4 y | 490 (264 - 912) |
|  |  |  |  | 5-14 y | 489 (338 - 709) |
|  |  |  |  | >14 y | 141 (82 - 243) |
|  | Madagascar | Imerintsiatosika | 2011 - 2013 | 0-1 y | 0 |
|  |  |  |  | 2-4 y | 0 |
|  |  |  |  | 5-14 y | 171 (81 - 360) |
|  |  |  |  | >14 y | 20 (4 - 103) |
|  |  | Isotry | 2012 - 2013 | 0-1 y | 0 |
|  |  |  |  | 2-4 y | 0 |
|  |  |  |  | 5-14 y | 62 (11 - 359) |
|  |  |  |  | >14 y | 42 (12 - 151) |
|  | Senegal | Pikine | 2011 - 2013 | 0-1 y | 0 |
|  |  |  |  | 2-4 y | 0 |
|  |  |  |  | 5-14 y | NA |
|  |  |  |  | >14 y | NA |
|  | South Africa | Pietermaritzburg | 2012 - 2014 | 0-1 y | 0 |
|  |  |  |  | 2-4 y | 0 |
|  |  |  |  | 5-14 y | 0 |
|  |  |  |  | >14 y | NA (19) |
|  | Sudan | East Wad Medani | 2012 - 2013 | 0-1 y | 0 |
|  |  |  |  | 2-4 y | 0 |
|  |  |  |  | 5-14 y | 0 |
|  |  |  |  | >14 y | 0 |
|  | Tanzania | Moshi Rural District | 2011 - 2013 | 0-1 y | 0 |
|  |  |  |  | 2-4 y | 0 |
|  |  |  |  | 5-14 y | 18 (8 - 44) |
|  |  |  |  | >14 y | 28 (8 - 95) |
|  | Tanzania | Moshi Urban District | 2011 - 2013 | 0-1 y | 0 |
|  |  |  |  | 2-4 y | 1028 (472 - 2237) |
|  |  |  |  | 5-14 y | 103 (54 - 199) |
|  |  |  |  | >14 y | 201 (99 - 408) |
| Marks [3] (2022) | Burkina Faso | Nioko and Polesgo | 2016 - 2019 | 0-1 y | 0 |
|  |  |  |  | 2-4 y | 1699 (188 - 6366) |
|  |  |  |  | 5-14 y | 2493 (546 - 8333) |
|  |  |  |  | >14 y | 779 (236 - 2201) |
|  | DR Congo | Kavuaya and Nkandu 1 | 2017 - 2020 | 0-1 y | 608 (90 - 3251) |
|  |  |  |  | 2-4 y | 507 (257 - 1079) |
|  |  |  |  | 5-14 y | 545 (423 - 694) |
|  |  |  |  | >14 y | 181 (130 - 252) |
|  | Ethiopia | Sodo | 2017 - 2019 | 0-1 y | 0 |
|  |  |  |  | 2-4 y | 0 |
|  |  |  |  | 5-14 y | 54 (19 - 178) |
|  |  |  |  | >14 y | 12 (3 - 40) |
|  | Ghana | Agogo | 2016 - 2019 | 0-1 y | 44 (10 - 162) |
|  |  |  |  | 2-4 y | 286 (152 - 570) |
|  |  |  |  | 5-14 y | 280 (227 - 358) |
|  |  |  |  | >14 y | 33 (16 - 103) |
|  | Madagascar | Imerintsiatosika | 2016 - 2019 | 0-1 y | 0 |
|  |  |  |  | 2-4 y | 84 (10 - 649) |
|  |  |  |  | 5-14 y | 263 (198 - 348) |
|  |  |  |  | >14 y | 177 (144 - 219) |
|  |  | Mahajanga | 2018 - 2019 | 0-1 y | 0 |
|  |  |  |  | 2-4 y | 0 |
|  |  |  |  | 5-14 y | 400 (34 - 2690) |
|  |  |  |  | >14 y | 0 |
|  | Nigeria | Ibadan | 2017 - 2019 | 0-1 y | 160 (4 - 947) |
|  |  |  |  | 2-4 y | 93 (41 - 189) |
|  |  |  |  | 5-14 y | 106 (82 - 139) |
|  |  |  |  | >14 y | 5 (2 - 11) |
| Meiring (2021) [4] | Malawi | Ndirande | 2016 - 2018 | 0-4 y | 632 (398 - 965) |
|  |  |  |  | 5-9 y | 861 (599 - 1203) |
|  |  |  |  | 10-14 y | 602 (377 - 915) |
|  |  |  |  | 15-29 y | 361 (219 - 567) |
|  |  |  |  | >30 y | 248 (124 - 447) |
| Thriemer (2012) [5] | Tanzania | Pemba Island | 2010 - 2010 | 0-5 y | 84 (69 - 101) |
|  |  |  |  | 6-15 y | 101 (86 - 121) |
|  |  |  |  | >15 y | 128 (115 - 143) |

^*^Incidence rates were not adjusted for blood culture sensitivity

**Table B** in S1 Information. Estimated coefficients of covariates in the proposed model, sub-Saharan Africa, 2017. Greyed cells indicate variables that were removed before modeling to reduce multicollinearity. Cells with the blue background indicate variables that were removed because the *p*-values were larger than or near to 0.05 and excluding them reduced the LOO cross-validation RMSE.

| Model | Variable | 0-1 y | | | 2-4 y | | | 5-14 y | | | >14 y | | |
| --- | --- | --- | --- | --- | --- | --- | --- | --- | --- | --- | --- | --- | --- |
|  |  | Est. | SE | *p*-value | Est. | SE | *p*-value | Est. | SE | *p*-value | Est. | SE | *p*-value |
| Pois | Improved water |  |  |  | 0.105 | 0.008 | < 0.001 | 0.024 | 0.005 | < 0.001 | 0.016 | 0.011 | 0.134 |
|  | Improved sanitation |  |  |  |  |  |  |  |  |  |  |  |  |
|  | Annual rainfall |  |  |  | -2.122 | 0.281 | < 0.001 | 0.422 | 0.068 | < 0.001 | 0.616 | 0.131 | 0.000 |
|  | Annual mean temperature |  |  |  |  |  |  |  |  |  |  |  |  |
|  | Stunting prevalence |  |  |  |  |  |  | 5.429 | 1.472 | < 0.001 | 8.393 | 2.212 | 0.000 |
|  | HIV prevalence |  |  |  | 0.185 | 0.027 | < 0.001 | 0.051 | 0.012 | < 0.001 | 0.023 | 0.013 | 0.092 |
|  | Travel time to the nearest city |  |  |  | 0.032 | 0.005 | < 0.001 | 0.005 | 0.002 | 0.001 |  |  |  |
|  | Elevation | -3.848 | 0.860 | < 0.001 |  |  |  | -0.468 | 0.218 | 0.032 | -0.803 | 0.398 | 0.043 |
|  | Distance to water | 0.082 | 0.017 | < 0.001 | 0.396 | 0.026 | < 0.001 | 0.095 | 0.010 | < 0.001 | 0.066 | 0.012 | 0.000 |
|  | Access to piped water |  |  |  | -0.026 | 0.005 | < 0.001 | 0.010 | 0.003 | < 0.001 | 0.018 | 0.006 | 0.001 |
|  | Access to piped sanitation | -0.112 | 0.024 | < 0.001 | 0.083 | 0.007 | < 0.001 | 0.025 | 0.003 | < 0.001 | 0.036 | 0.005 | < 0.001 |
|  | Access to surface water | 0.079 | 0.015 | < 0.001 | 0.232 | 0.014 | < 0.001 | 0.057 | 0.007 | < 0.001 | 0.088 | 0.012 | < 0.001 |
|  | Open defecation | 0.068 | 0.014 | < 0.001 | 0.037 | 0.007 | < 0.001 | 0.012 | 0.004 | 0.001 | 0.012 | 0.006 | 0.054 |
|  | Wasting | -70.458 | 11.988 | < 0.001 | 65.145 | 5.004 | < 0.001 | 19.545 | 2.227 | < 0.001 |  |  |  |
|  | Underweight |  |  |  |  |  |  |  |  |  |  |  |  |
|  | Population density | 1.312 | 0.210 | 0.000 | 0.920 | 0.088 | 0.000 | 0.506 | 0.062 | 0.000 | 0.376 | 0.090 | < 0.001 |
| NegBin | Improved water |  |  |  | 0.034 | 0.023 | 0.149 |  |  |  |  |  |  |
|  | Improved sanitation |  |  |  |  |  |  |  |  |  |  |  |  |
|  | Annual rainfall |  |  |  |  |  |  | 0.343 | 0.207 | 0.098 | 0.691 | 0.238 | 0.004 |
|  | Annual mean temperature |  |  |  |  |  |  |  |  |  |  |  |  |
|  | Stunting prevalence |  |  |  |  |  |  |  |  |  | 11.935 | 4.332 | 0.006 |
|  | HIV prevalence |  |  |  |  |  |  |  |  |  |  |  |  |
|  | Travel time to the nearest city |  |  |  |  |  |  |  |  |  |  |  |  |
|  | Elevation | -5.105 | 2.072 | 0.014 |  |  |  |  |  |  | -1.151 | 0.694 | 0.097 |
|  | Distance to water | 0.144 | 0.050 | 0.004 | 0.088 | 0.042 | 0.038 | 0.064 | 0.022 | 0.004 | 0.101 | 0.028 | 0.000 |
|  | Access to piped water |  |  |  |  |  |  | 0.029 | 0.008 | 0.000 | 0.043 | 0.013 | 0.001 |
|  | Access to piped sanitation | -0.139 | 0.057 | 0.016 |  |  |  |  |  |  | 0.028 | 0.013 | 0.029 |
|  | Access to surface water | 0.092 | 0.043 | 0.033 | 0.080 | 0.039 | 0.040 | 0.036 | 0.018 | 0.049 | 0.104 | 0.024 | 0.000 |
|  | Open defecation | 0.067 | 0.035 | 0.053 |  |  |  |  |  |  |  |  |  |
|  | Wasting | -88.002 | 30.004 | 0.003 |  |  |  | 11.518 | 6.003 | 0.055 |  |  |  |
|  | Underweight |  |  |  |  |  |  |  |  |  |  |  |  |
|  | Population density | 1.353 | 0.505 | 0.007 | 0.398 | 0.259 | 0.125 |  |  |  | 0.347 | 0.195 | 0.075 |

Pois = Poisson regression; NegBin = Negative Binomial regression; Est = Estimate; SE = Standard error

**Section A** in S1 Information. Catchment area for the incidence rates

Polygons that represent the catchment area of each surveillance site were created in the shapefile format during surveillance in Typhoid Fever Surveillance Program in Africa (TSAP) [2] and Severe Typhoid Fever in Africa (SETA) programs [6]. These shapefiles were used to extract geospatial covariates that correspond to the catchment areas. Values of the geospatial covariates were extracted using the ‘extract’ function of the raster package in R. The ‘extract’ function returns the values of the grid cells of the geospatial covariates that are covered by a polygon. A grid cell is covered by a polygon if its center is inside the polygon. If a polygon covers multiple grids, we took the mean to create a single representative value.

## Fig A in S1 Information. Catchment area represented as 20 km × 20 km grids. Shapefiles specific to the African continent are available for download from GADM at: https://gadm.org/license.html.


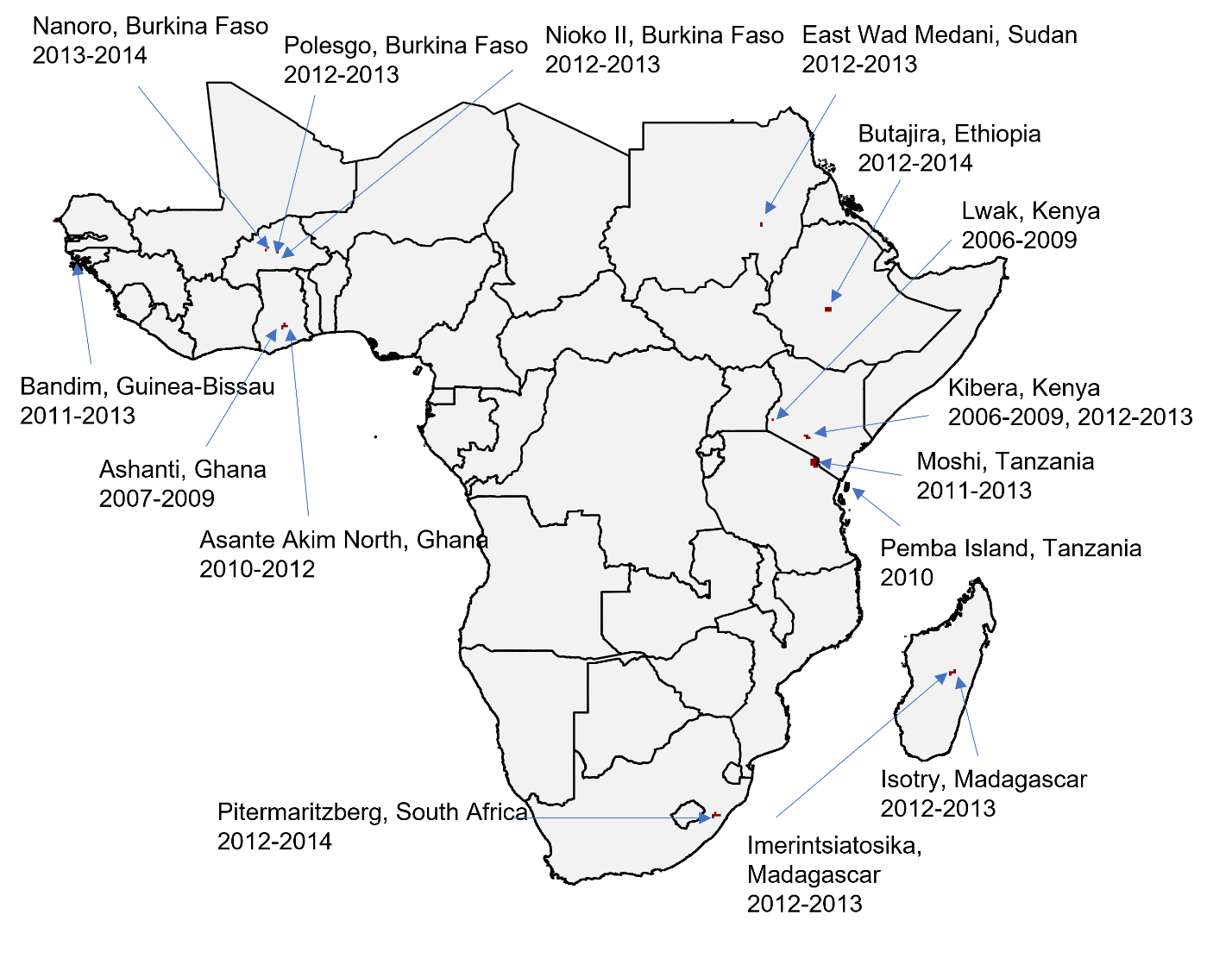


## Fig B in S1 Information. Percentage access to improved sanitation facilities based on Deshpande *et* *al*. [7]. The improved sanitation includes sewer or septic tanks and other improved sanitation facilities (improved latrines, ventilated improved latrines, composting toilets). The dataset is available at the IHME: https://cloud.ihme.washington.edu/s/bkH2X2tFQMejMxy.


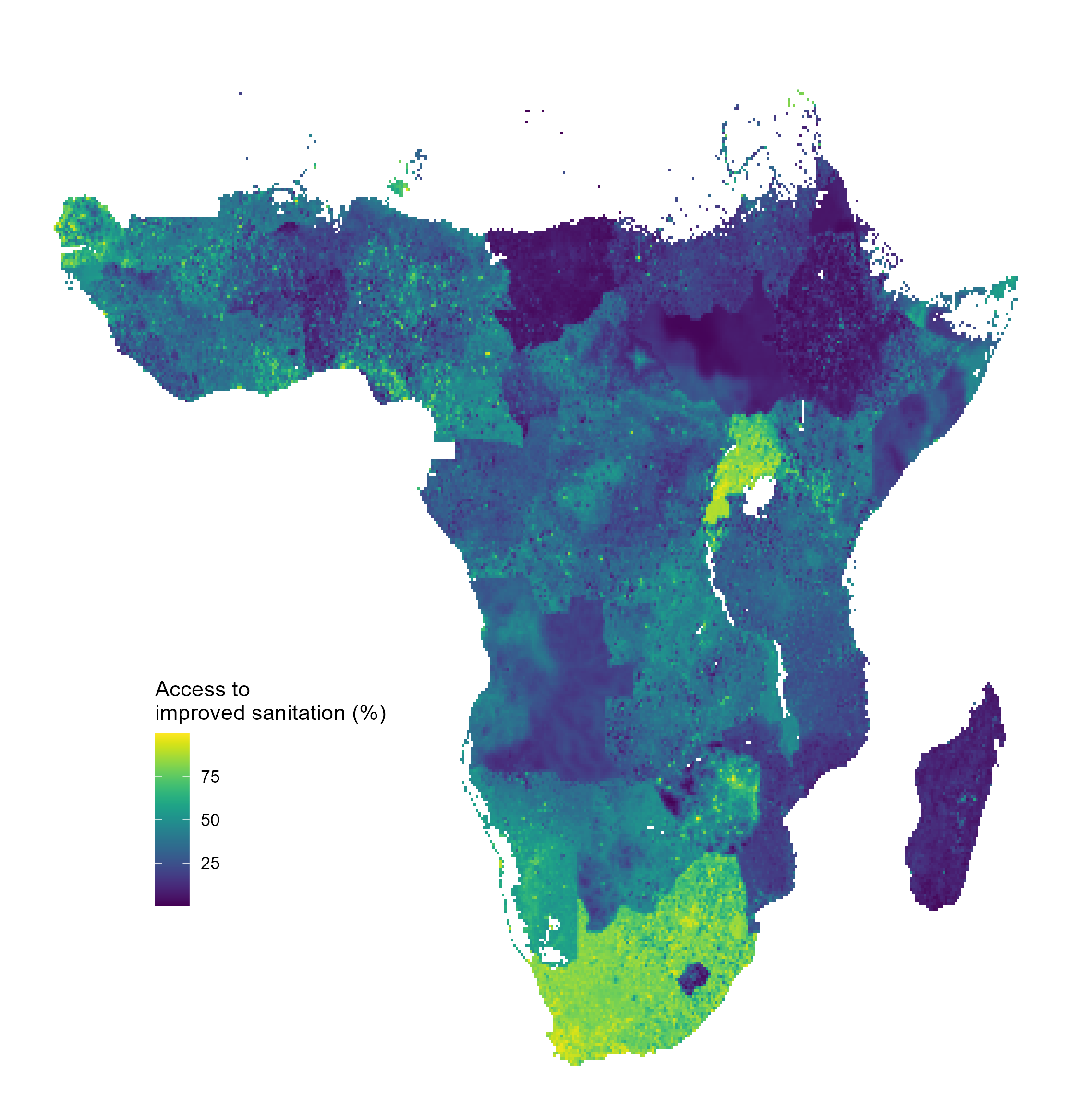


## Fig C in S1 Information. Percentage access to sewer or septic sanitation facilities based on Deshpande *et* *al*. [7]. The dataset is available at the IHME: ttps://cloud.ihme.washington.edu/s/bkH2X2tFQMejMxy.


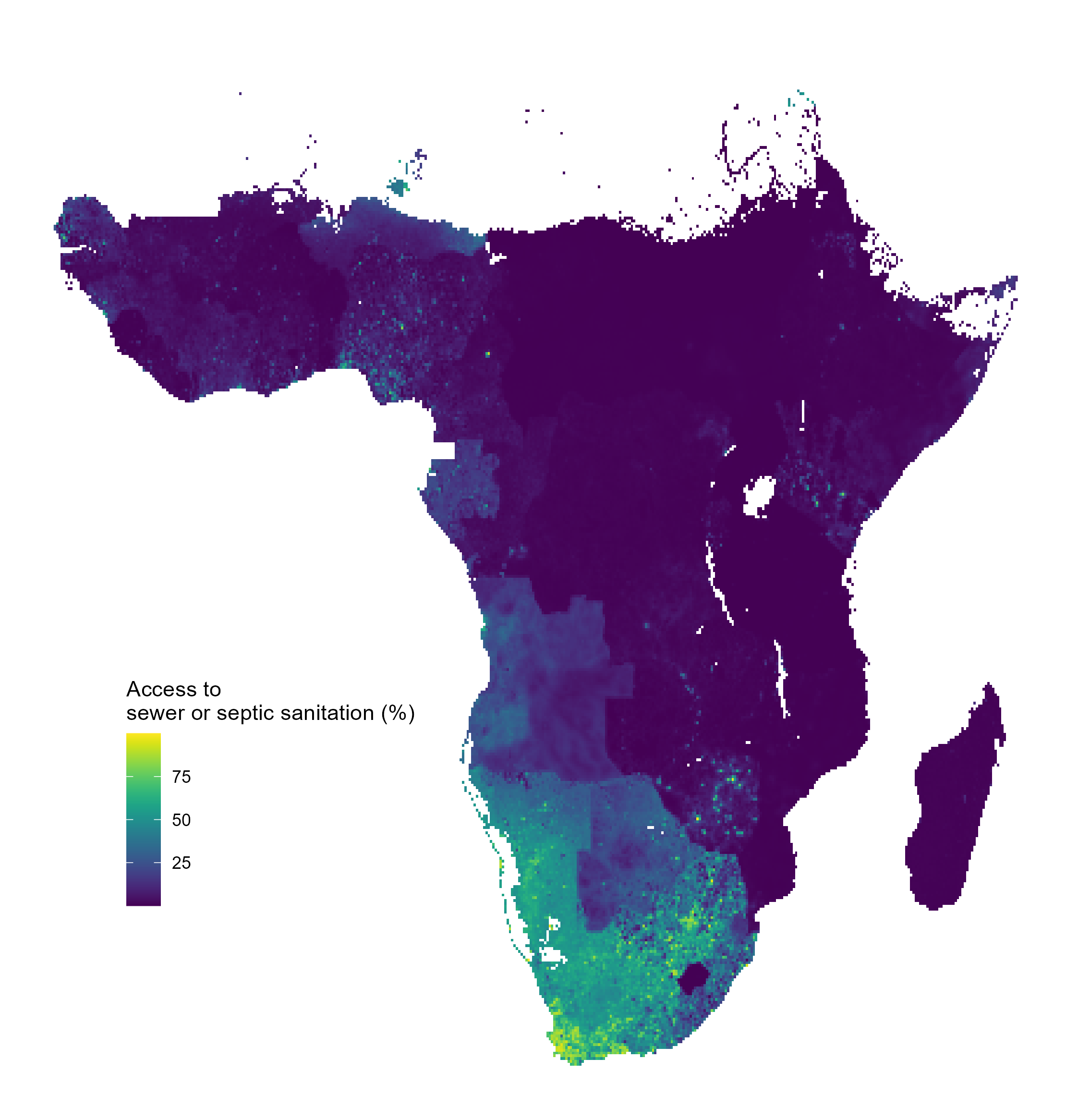


## Fig D in S1 Information. Percentage open defecation based on Deshpande *et* *al*. [7]. The dataset is available at the IHME: https://cloud.ihme.washington.edu/s/bkH2X2tFQMejMxy.


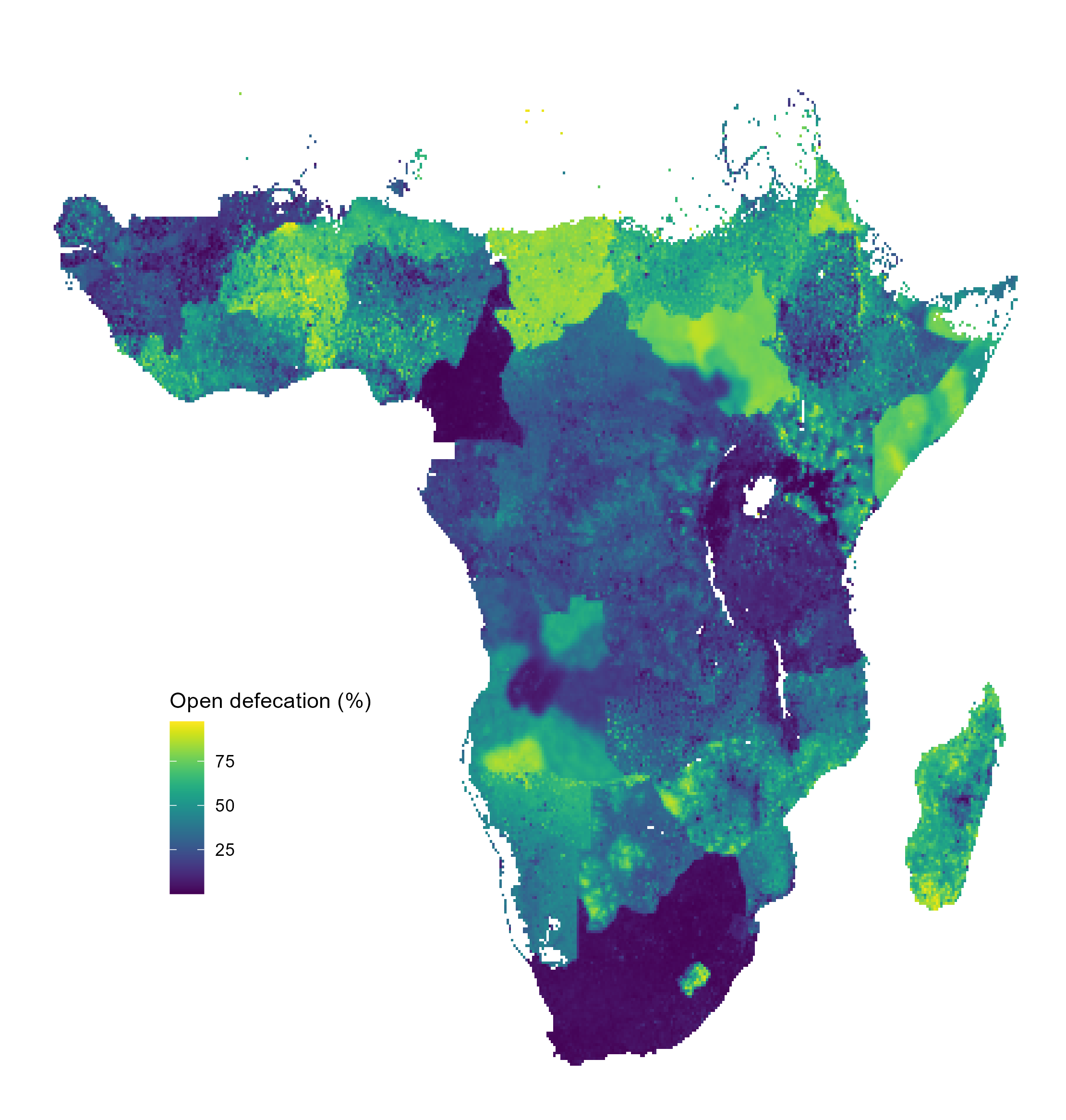


## Fig E in S1 Information. Access to improved drinking water based on Deshpande *et* *al*. [7] The improved water indicates access to piped water according to the JMP definition and includes piped (piped on or off premises) and other improved (protected wells and springs, bottled water, rainwater collection, bought water) water. The dataset is available at the IHME: https://cloud.ihme.washington.edu/s/bkH2X2tFQMejMxy.


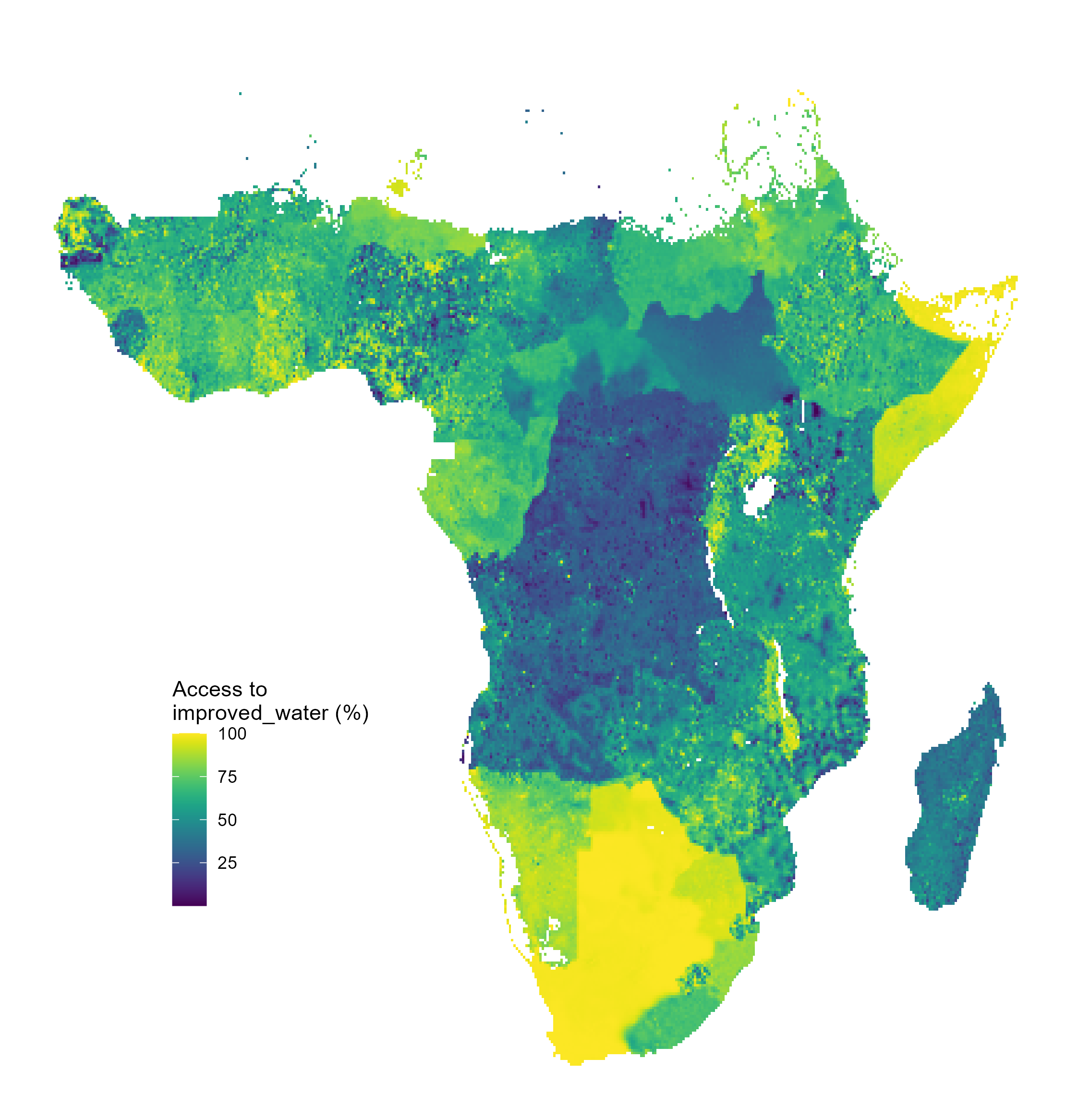


## Fig F in S1 Information. Access to piped drinking water based on Deshpande *et* *al*. [7]. The dataset is available at the IHME: https://cloud.ihme.washington.edu/s/bkH2X2tFQMejMxy.


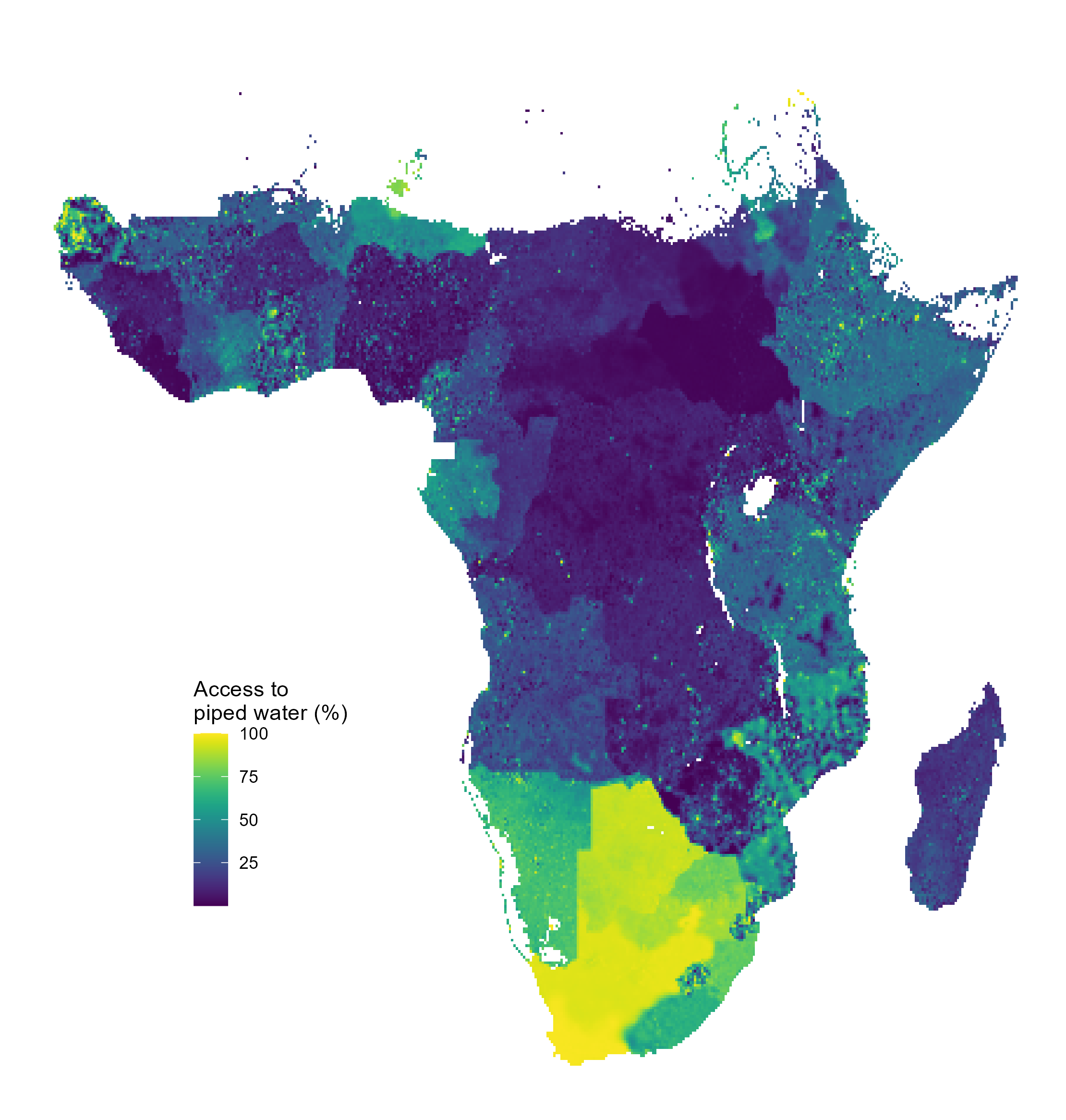


## Fig G in S1 Information. Use of surface water based on Deshpande *et* *al*. [7]. The dataset is available at the IHME: https://cloud.ihme.washington.edu/s/bkH2X2tFQMejMxy.


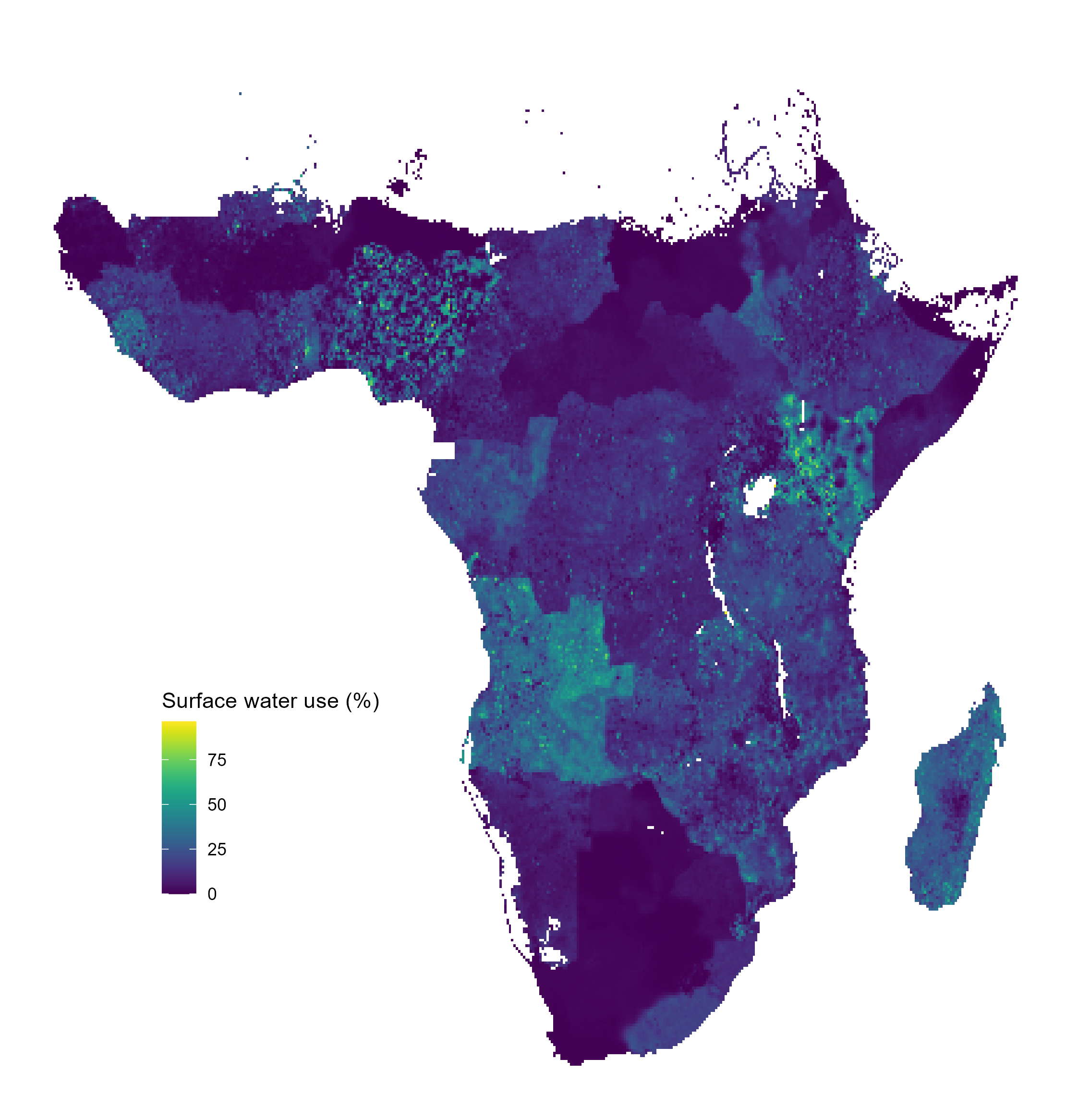


## Fig H in S1 Information. Precipitation. The values indicate annual precipitation summed across daily precipitation data Climate Hazards group Infrared Precipitation with Stations (CHIRPS) data set [8]. The dataset is available at https://cds.climate.copernicus.eu/cdsapp#!/dataset/insitu-gridded-observations-global-and-regional?tab=form. Shapefiles specific to the African continent are available for download from GADM at: https://gadm.org/license.html.


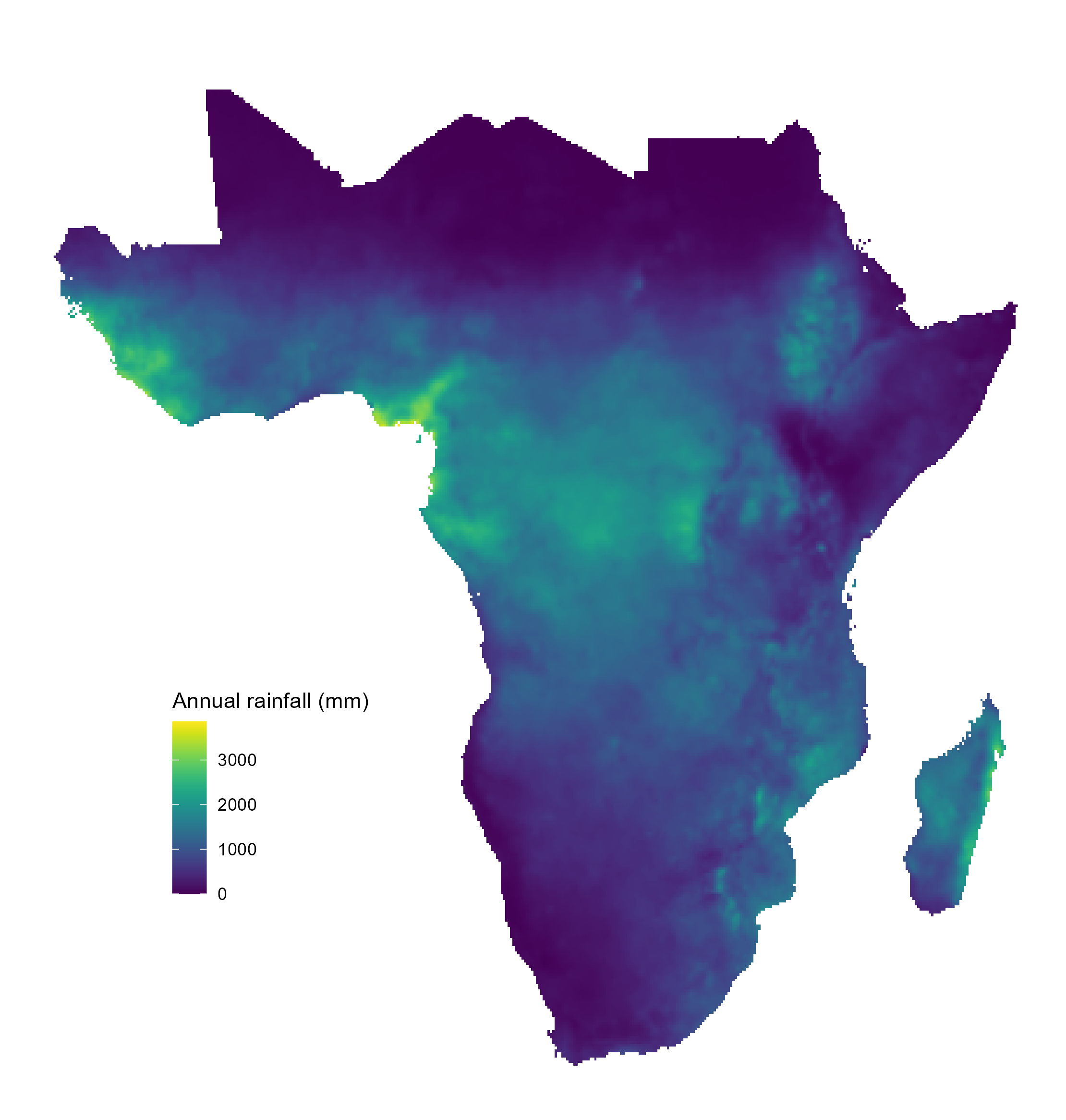


## Fig I in S1 Information. Annual mean temperature for 2017. Monthly mean temperature [8] were averaged. The dataset is available at https://cds.climate.copernicus.eu/cdsapp#!/dataset/insitu-gridded-observations-global-and-regional?tab=form. Shapefiles specific to the African continent are available for download from GADM at: https://gadm.org/license.html.


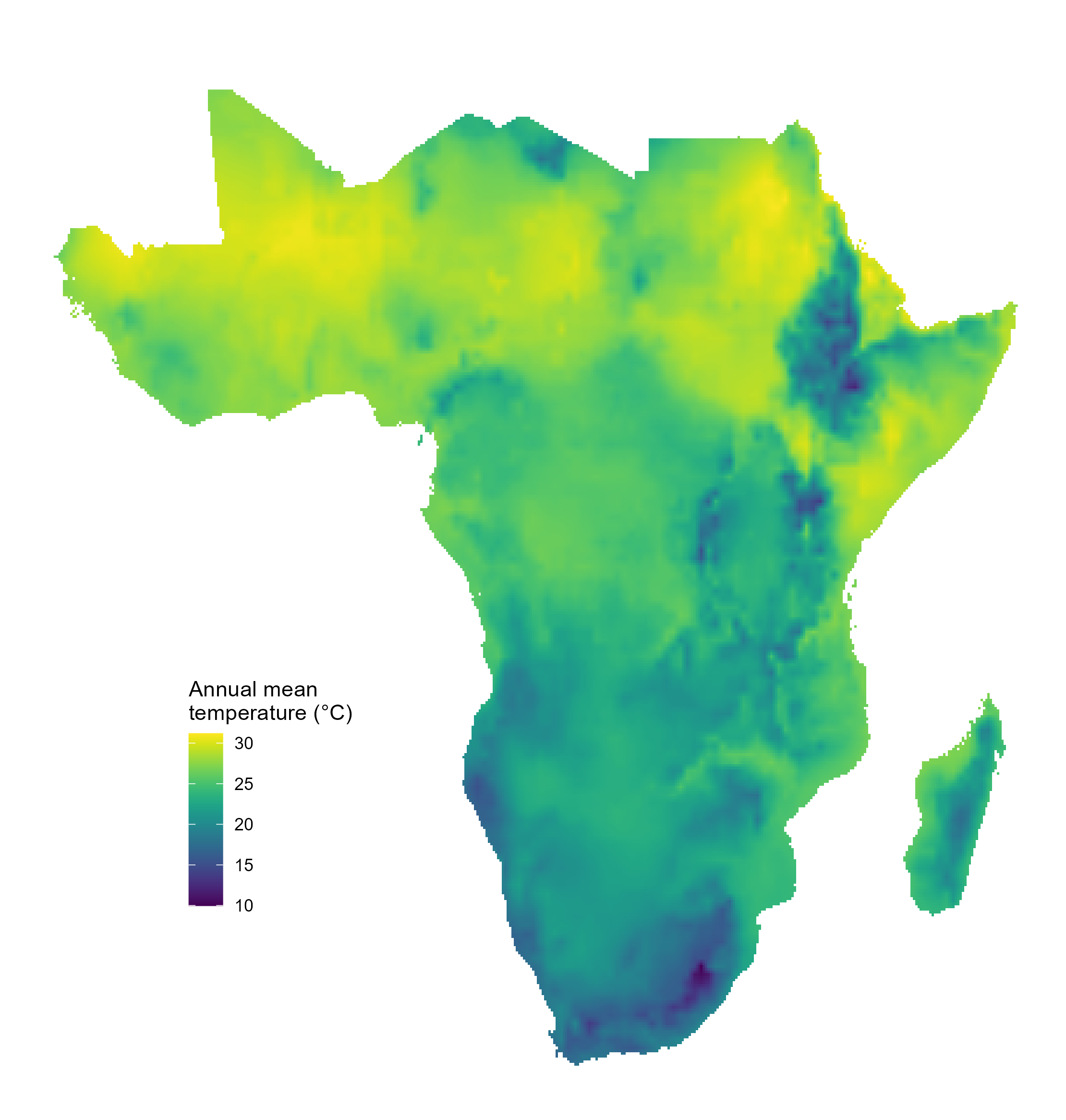


## Fig J in S1 Information. Prevalence of stunting among children under the age of 5 in Africa for 2017 based on the study by Kinyoki *et* *al*.[9]. The dataset is available at <https://cloud.ihme.washington.edu/index.php/s/Q5CGeazb4iNsDQA>. Shapefiles specific to the African continent are available for download from GADM at: https://gadm.org/license.html.


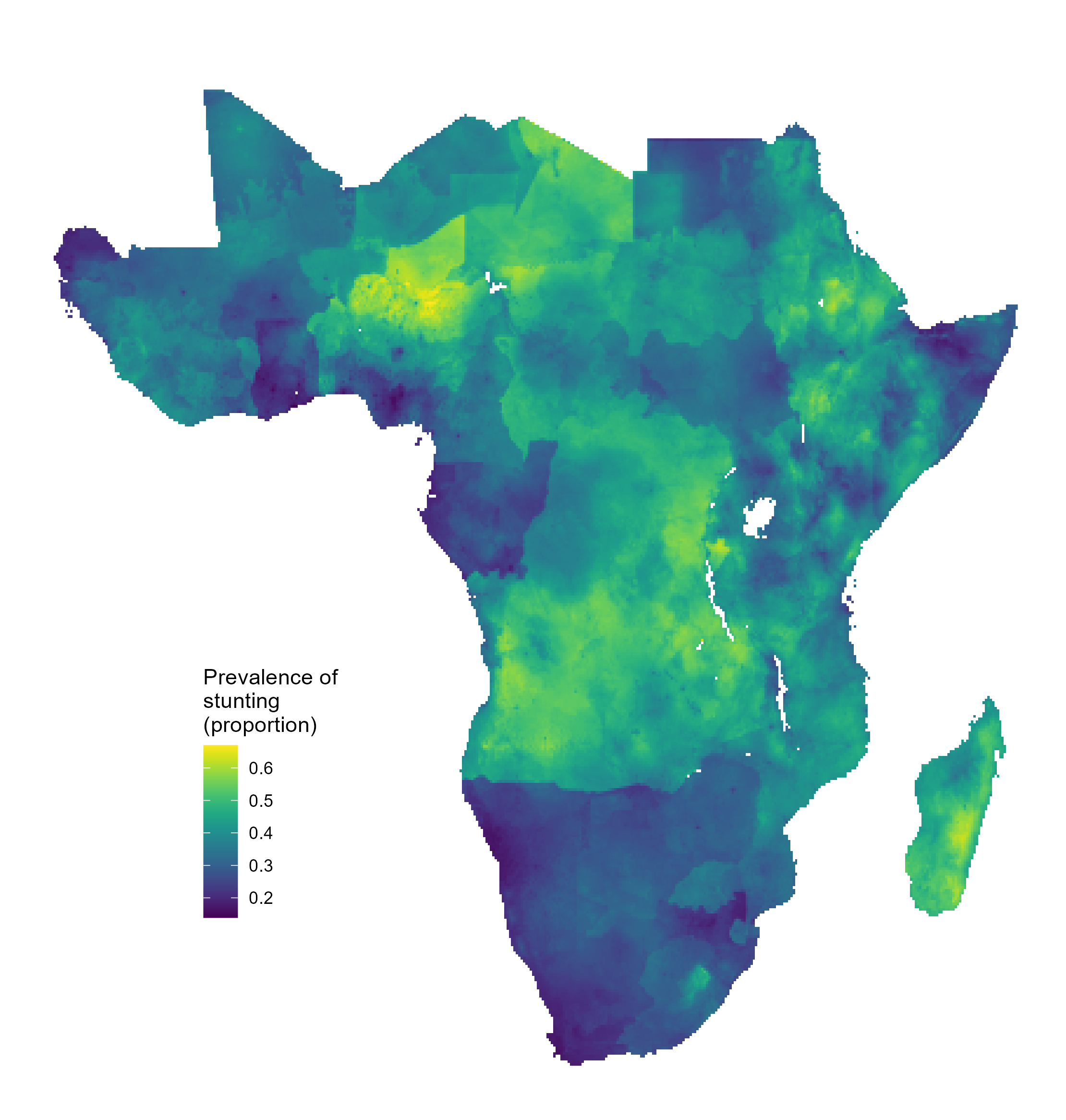


## Fig K in S1 Information. Prevalence of wasting among children under the age of 5 in Africa for 2017 based on the study by Kinyoki *et* *al*.[9]. The dataset is available at https://cloud.ihme.washington.edu/index.php/s/Q5CGeazb4iNsDQA. Shapefiles specific to the African continent are available for download from GADM at: https://gadm.org/license.html.


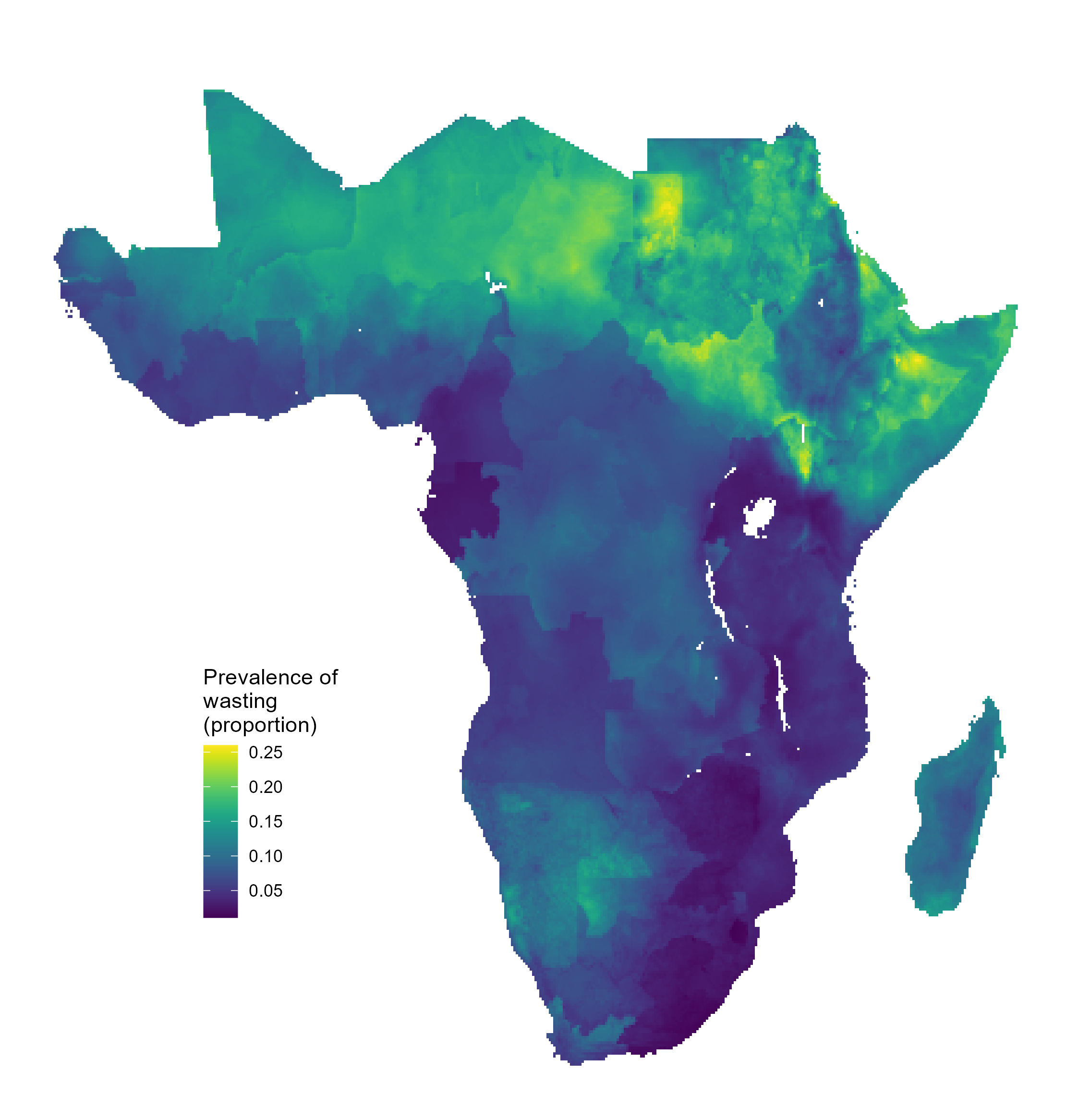


## Fig L in S1 Information. Prevalence of underweight among children under the age of 5 in Africa for 2017 based on the study by Kinyoki *et* *al*.[9]. The dataset is available at <https://cloud.ihme.washington.edu/index.php/s/Q5CGeazb4iNsDQA>. Shapefiles specific to the African continent are available for download from GADM at: https://gadm.org/license.html.

**
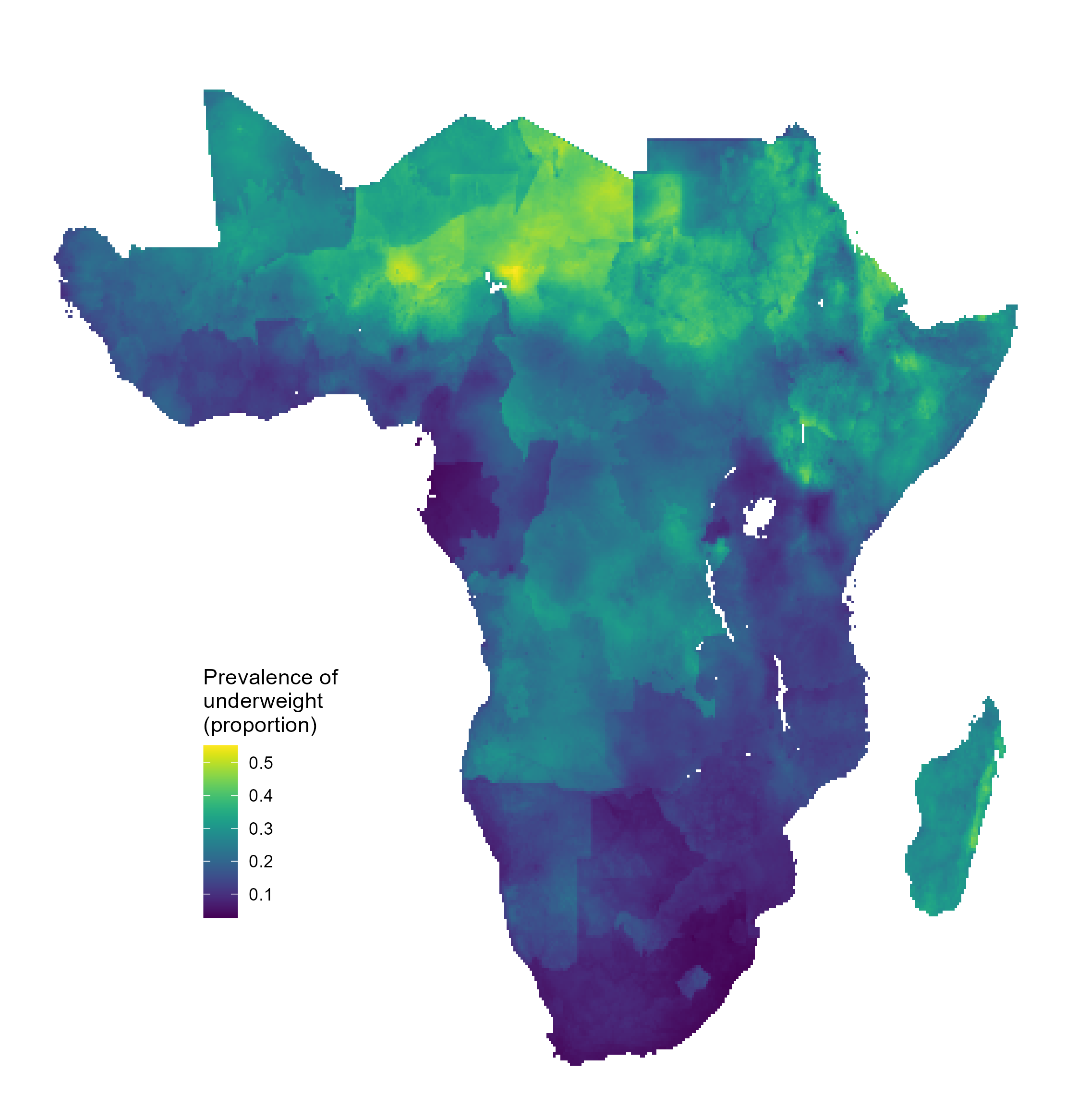
**

**Fig M** in S1 Information. Prevalence of HIV infection among adults (15-49 years old) in sub-Saharan Africa for 2017 based on the study by Dwyer-Lindgren *et* *al*.[10]. The dataset is available at https://ghdx.healthdata.org/record/ihme-data/africa-hiv-prevalence-geospatial-estimates-2000-2017. Shapefiles specific to the African continent are available for download from GADM at: https://gadm.org/license.html.


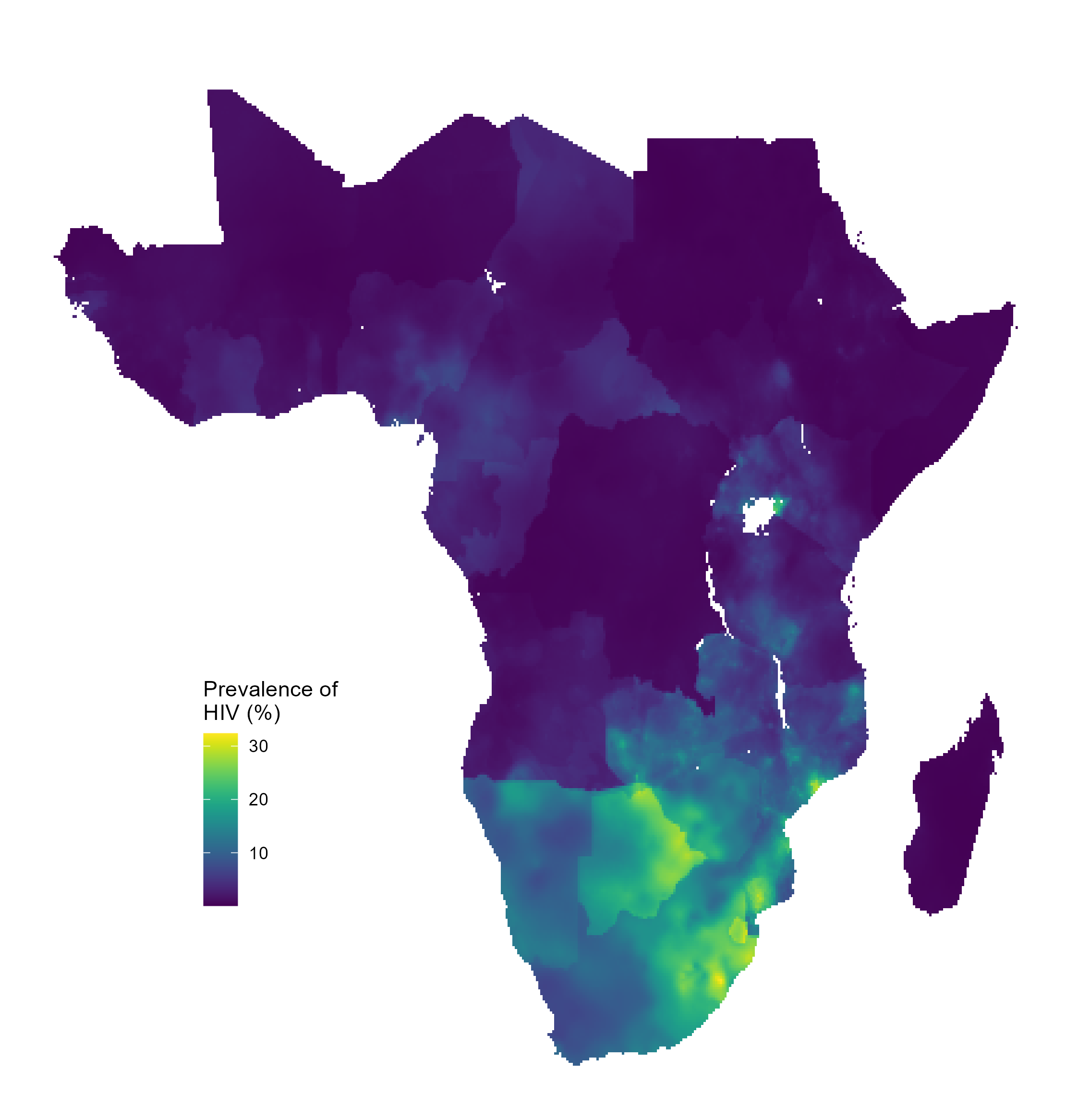


## Fig N in S1 Information. Travel time to cities (in minutes) in Africa for 2017 based on the study by Weiss *et* *al*.[11]. The dataset is available at <https://data.malariaatlas.org/maps>. Shapefiles specific to the African continent are available for download from GADM at: https://gadm.org/license.html.


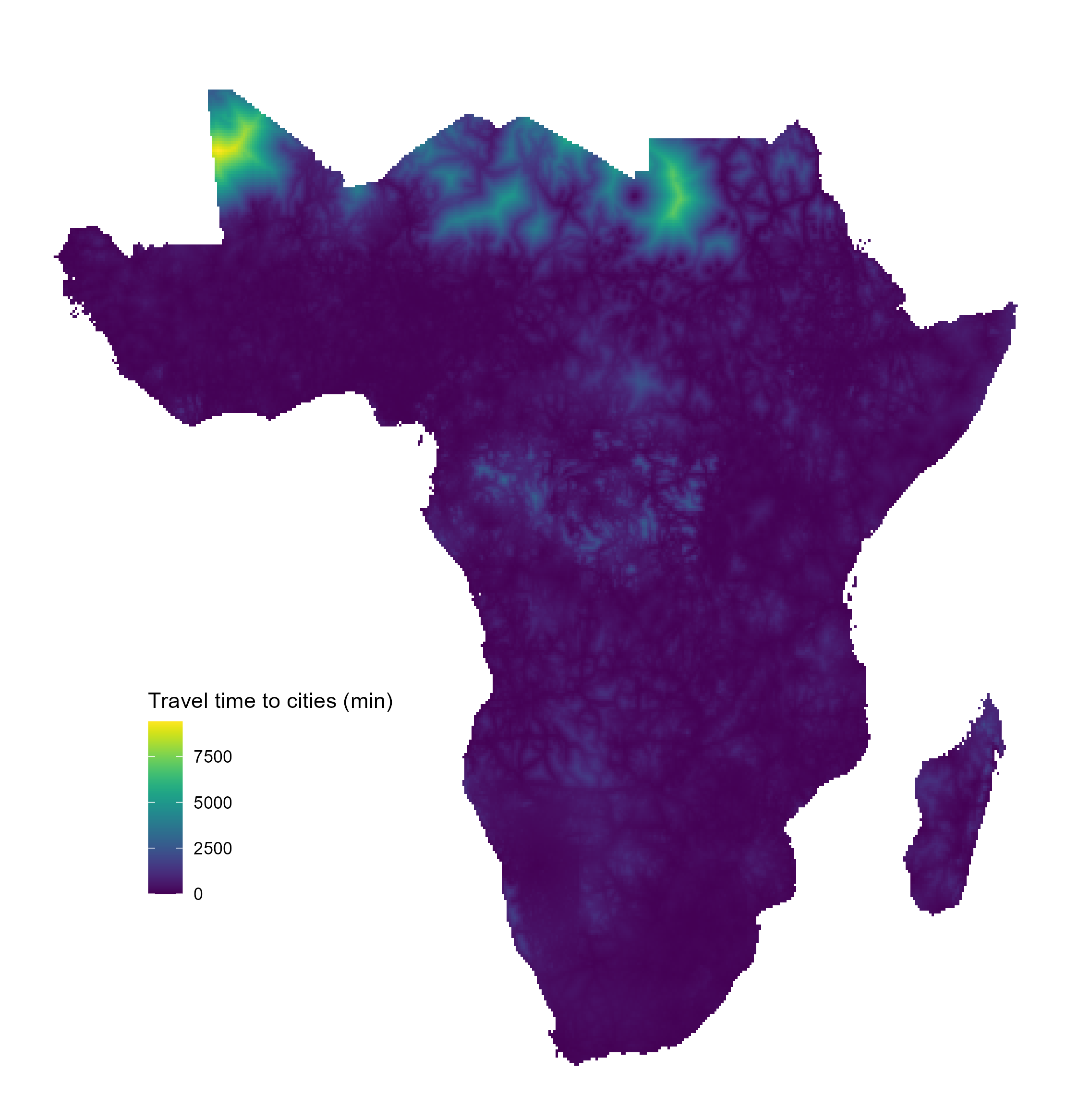


## Fig O in S1 Information. Distance to water. The value indicates the distance for each pixel to the nearest water cell (inland and sea) at 20 km × 20 km resolution. For the water cell the distance to water is 0 km.[12]. The dataset is available at <https://data.ceda.ac.uk/neodc/globolakes/data/v1/limnology>. Shapefiles specific to the African continent are available for download from GADM at: https://gadm.org/license.html.

**
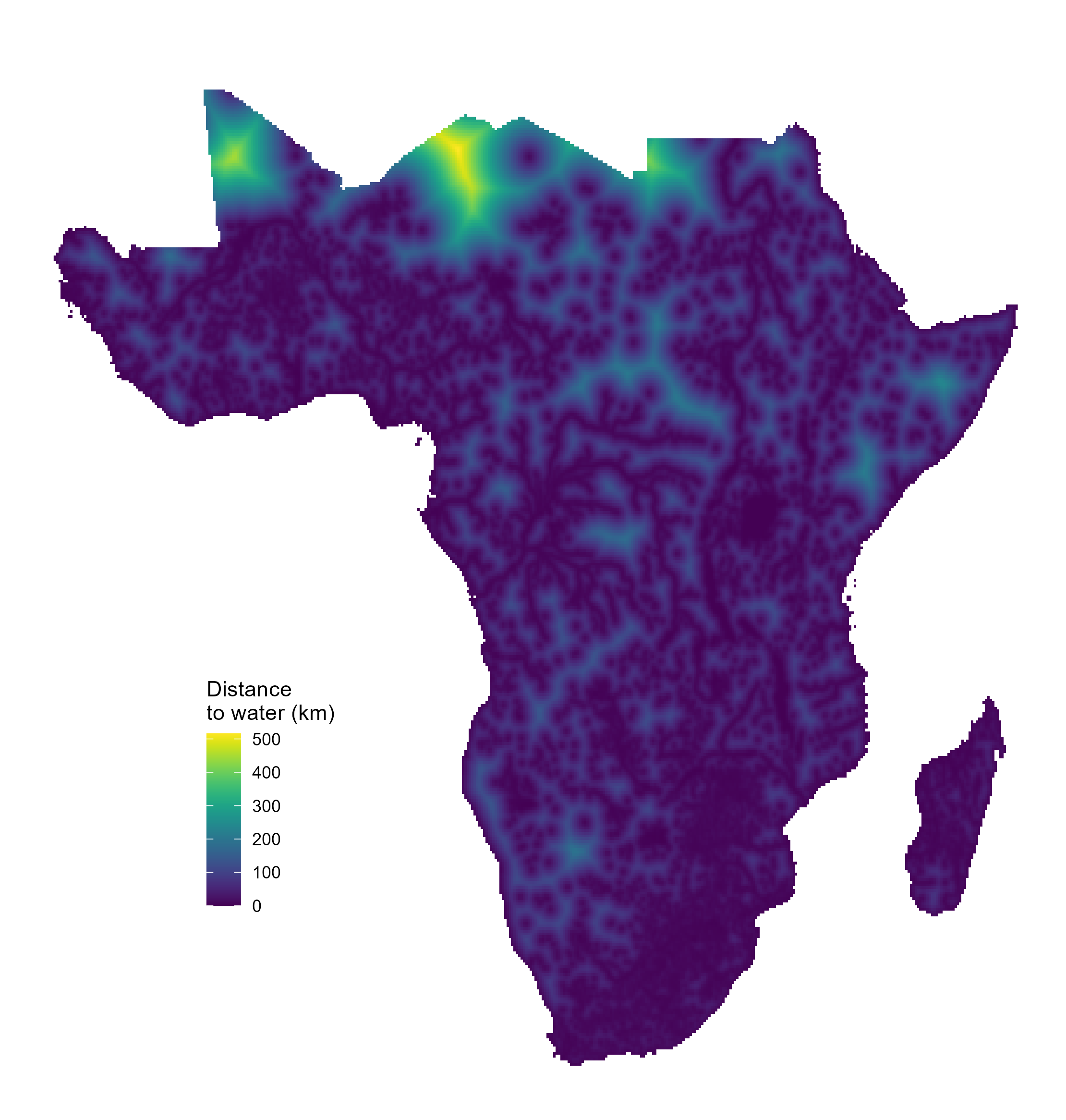
**

Fig P in S1 Information. Elevation. Elevation data come from estimates by Mapzen that combine several digital elevation model (DEM) such as the Shuttle Radar Topography Mission (SRTM), the USGS National Elevation Dataset (NED), Global DEM (GDEM), and others. R package ‘elevatr’ serves as an API that enables an access to the elevation estimates by Mapzen hosted at Amazon Web Services Terrain Tiles.[13]. Shapefiles specific to the African continent are available for download from GADM at: https://gadm.org/license.html.


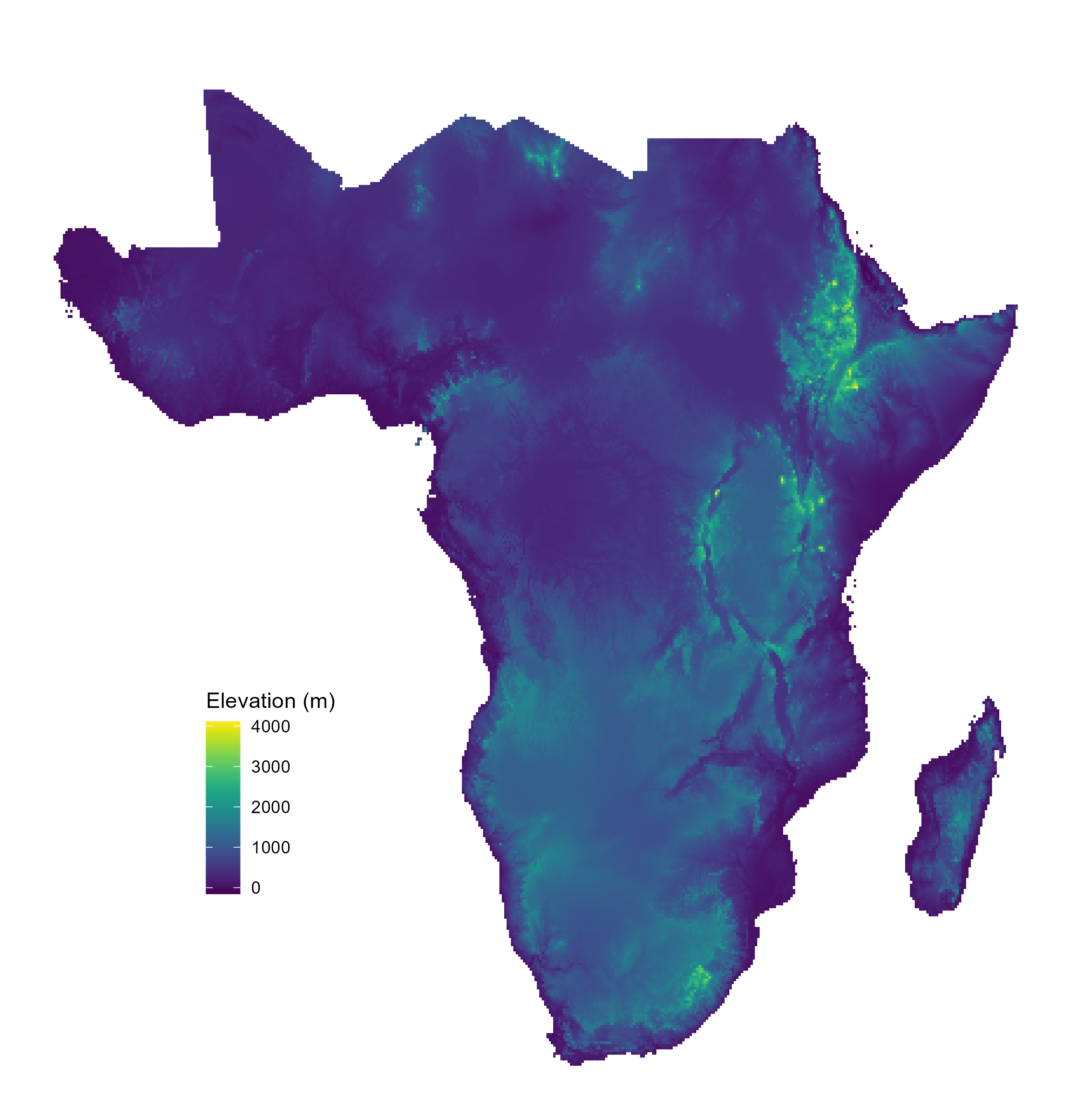


Fig Q in S1 Information. Population count per pixel based on the WorldPop [14]. Mosaiced 1km resolution global dataset were aggregated to create 20 km resolution dataset. The dataset is available at <https://hub.worldpop.org/geodata/listing?id=64>. Shapefiles specific to the African continent are available for download from GADM at: https://gadm.org/license.html.


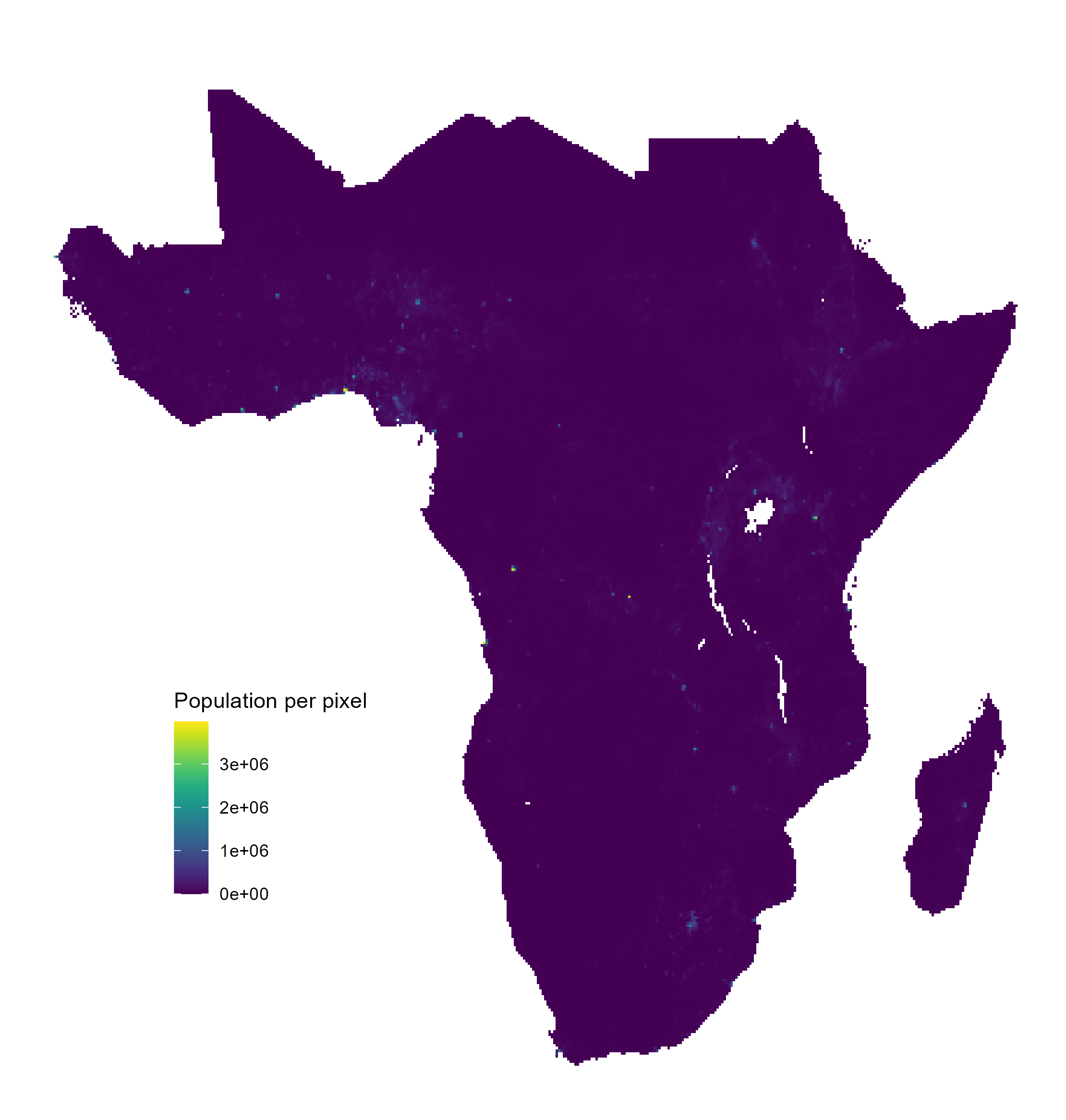


**Section B** in S1 Information. Multivariate regression for Poisson and negative binomial models

Let $Y_{a,i}$ be the observed incidence rate per 100,000 person years of a particular age class *a* (0-1, 2-4, 5-14, and 15+) at grid *i (=1,2,*$\cdots$*,N)*. We assumed that $Z_{a,i}=Y_{a,i}/10$ follows a Poisson or negative binomial distribution with probability mass function:

$$f\left( z_{a,i};\mu_{a,i} \right)=\frac{{\mu_{a,i}}^{z_{a,i}}e^{-\mu_{a,i}}}{z_{a,i}!}$$

or

$$f\left( z_{a,i};\mu_{a,i},\theta_{a} \right)=\frac{\Gamma({z_{a,i}+\theta}_{a})}{\Gamma\left( \theta_{a} \right)z_{a,i}!}\frac{{\mu_{a,i}}^{z_{a,i}}{\theta_{a}}^{\theta_{a}}}{{({\mu_{a,i}+\theta}_{a})}^{{z_{a,i}+\theta}_{a}}}$$

where $\mu_{a,i}$ is the true underlying mean of $Z_{a,i}$. For the Poisson distribution, the mean and variance are the same. For the negative binomial distribution, $\theta_{a}$ is the shape parameter for age group *a*, $\Gamma\left( \cdot\right)$ is the Gamma function, and the variance of $Z_{a,i}$ is ${Var(Z}_{a,i})=\mu_{a,i}+ \frac{{\mu_{a,i}}^{2}}{\theta_{a}}.$ The log-transformation of $\mu_{a,i}$ is modeled as a linear combination of the intercept $\beta_{0,a}$ and covariates $x_{j,i}$ (*j=1,2,*$\cdots$*,J)* with corresponding coefficients $\beta_{j,a}:$

$$\log\left( \mu_{a,i} \right)=\beta_{0,a}+\beta_{1,a}x_{1,i}+\beta_{2,a}x_{2,i}+\cdots+\beta_{J,a}x_{J,i}.$$

**Section C** in S1 Information. Model validation

Predicted incidence rates were validated with a leave-one-out cross-validation method.[15] The model was fitted to the sample data excluding one observation, and the fitted model was then used to predict the value of the excluded observation. The validation process was repeated *N* times for the data of size *N*. Predictive performance of the proposed model was assessed with the root mean-square error (RMSE):

$$\mathrm{RMSE}_{a}=\sqrt{\frac{1}{N}\sum_{i=1}^{N} \left( Y_{a, i}-\hat{Y}_{a, i}^{\left( -i \right)} \right)^{2}}$$

where $\mathbf{RMSE}_{\boldsymbol{a}}$ is the RMSE of the age group *a*, ${\hat{\boldsymbol{Y}}}_{\boldsymbol{a, i}}^{\left( \boldsymbol{-i} \right)}$ is the predicted incidence rate for age group *a* at location *i*, obtained from the model fitted to the data without the observation *i*.

**Table C** in S1 Information. The root mean squared error (RMSE) values of the proposed model (linear regression model) with the competing models (Poisson and Negative Binomial regression models)

| Models | 0-1 yo | 2-4 yo | 5-14 yo | >14 yo |
| --- | --- | --- | --- | --- |
| Log-linear regression model | 2472.5 | 2966.36 | 763.9 | 161.8 |
| Poisson regression model | 2345.6 | 2952.6 | 2117.5 | 2077.4 |
| Negative binomial regression model | 2965.2 | 920.7 | 406.7 | 217.8 |

**Fig R** in S1 Information. Distribution of predicted incidence rates per 100,000 person years by age (A) and incidence rate ratio (B) with children aged 5-14 years as a reference group. Incidence rates were summarized at subnational levels. Red dots indicate observed incidence rates.


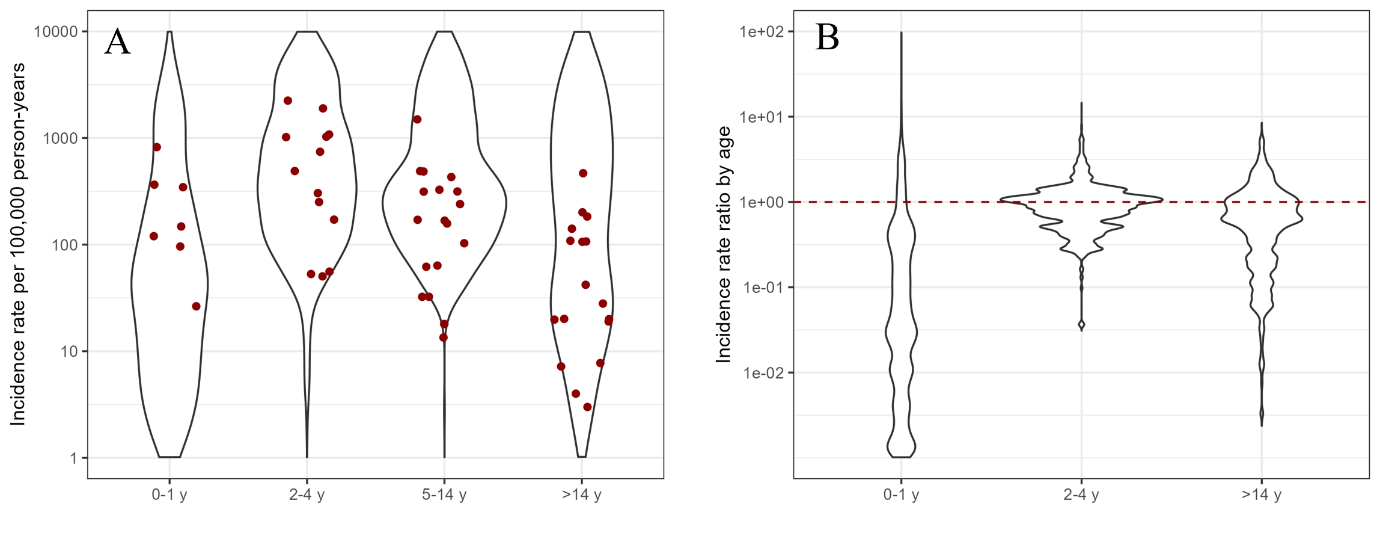


**Fig S** in S1 Information. Predicted incidence rates per 100,000 person-years for 0-1 yo (A), 2-4 yo (B), 5-14 yo (C), and >14 yo (D) summarized at country level. Bold and thin lines inside Africa represent country borders and first-level administrative divisions, respectively. Shapefiles specific to the African continent are available for download from GADM at: https://gadm.org/license.html.

**
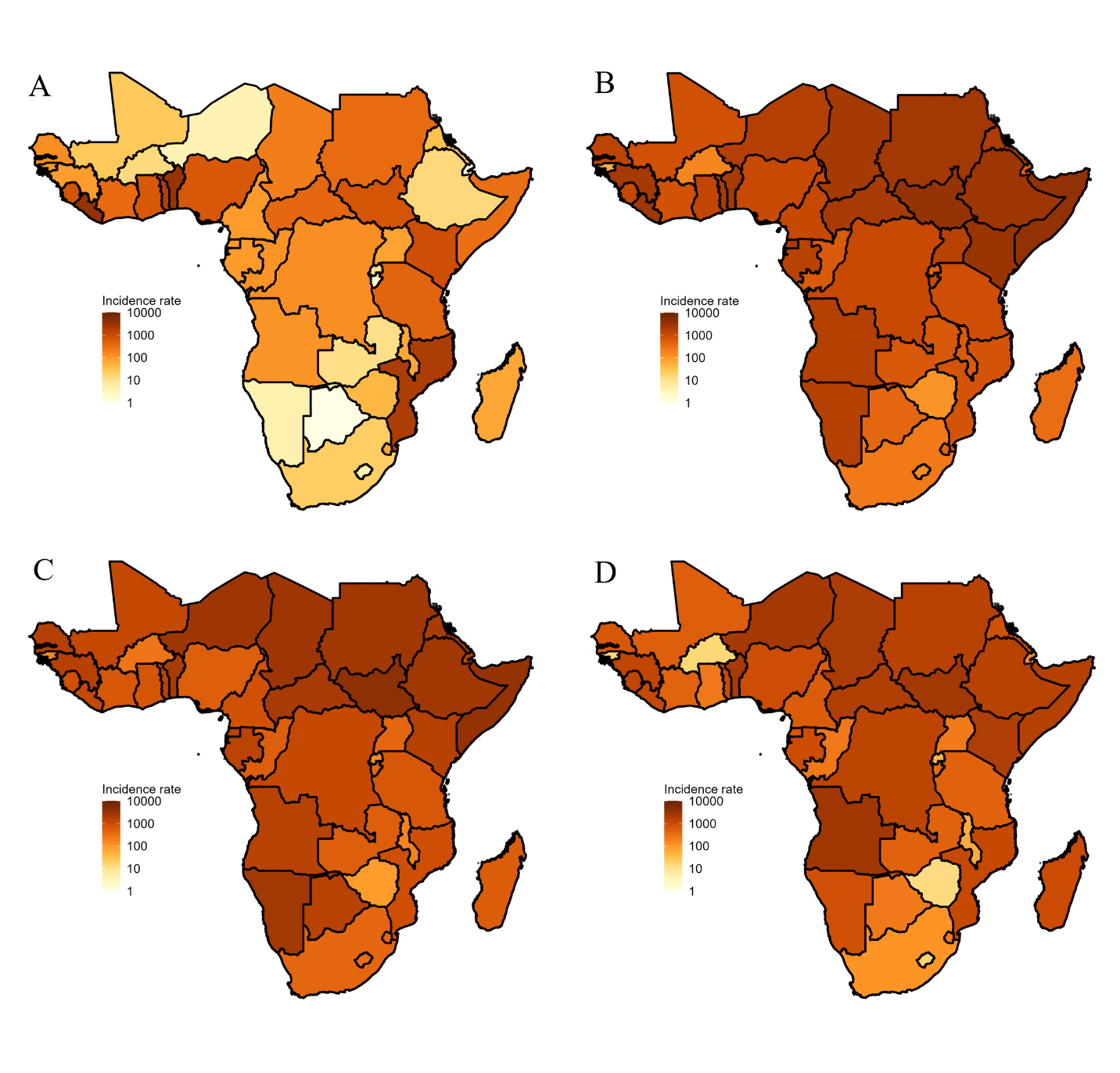
**

**Table D** in S1 Information. Estimated incidence rates per 100,000 person years by country, sub-Saharan Africa, 2017

| Country | Overall | 0-1 yo | 2-4 yo | 5-14 yo | > 14 yo |
| --- | --- | --- | --- | --- | --- |
| Angola | 2,050.4 (62.6 - 4,192.5) | 110.5 (25.8 - 452.3) | 1,708.6 (0 - 5,611.0) | 1,548.3 (4.3 - 4,189.4) | 2,641.5 (109.4 - 4,442.7) |
| Burundi | 445.2 (1.0 - 1,959.2) | 1.1 (0.0 - 11.6) | 1,101.1 (0 - 5,309.7) | 411.3 (0.0 - 2,113.7) | 388.8 (1.9 - 1,442.2) |
| Benin | 2,320.3 (103.6 - 3,653.8) | 3,188.6 (1,261.8 - 4,958.7) | 2,321.8 (0 - 5,473.2) | 2,242.8 (3.0 - 4,029.4) | 2,247.1 (22.8 - 3,008.8) |
| Burkina Faso | 100.3 (0.2 - 529.1) | 12.9 (2.2 - 60.3) | 166.9 (0 - 1,750.7) | 266.8 (0.0 - 1,016.4) | 14.5 (0.0 - 115.8) |
| Botswana | 523.4 (9.5 - 2,809.3) | 0.1 (0.0 - 1.0) | 414.6 (0 - 3,806.5) | 1,559.0 (5.2 - 4,731.8) | 234.2 (12.6 - 2,279.2) |
| CAR | 1,928.0 (13.2 - 3,219.7) | 401.1 (67.6 - 1,057.0) | 2,223.4 (0 - 4,306.6) | 2,223.8 (1.8 - 3,820.1) | 1,915.1 (14.7 - 2,956.2) |
| Cote d'Ivoire | 557.6 (10.9 - 1,780.8) | 376.8 (135.4 - 807.6) | 800.4 (0 - 3,698.7) | 688.9 (0.7 - 2,472.5) | 476.4 (3.2 - 1,251.7) |
| Cameroon | 653.7 (4.6 - 2,088.9) | 92.4 (10.0 - 497.6) | 1,129.2 (0 - 4,404.3) | 826.1 (0.9 - 2,730.8) | 562.4 (6.4 - 1,597.6) |
| DR Congo | 1,189.3 (7.5 - 2,482.5) | 133.3 (20.2 - 501.5) | 1,114.8 (0 - 3,694.8) | 1,142.2 (0.9 - 2,932.4) | 1,384.2 (10.5 - 2,301.3) |
| Congo | 351.3 (4.8 - 1,087.1) | 114.8 (21.9 - 290.6) | 623.2 (0 - 3,172.4) | 535.5 (0.7 - 1,452.4) | 247.3 (5.6 - 658.4) |
| Djibouti | 400.8 (0.6 - 1,459.6) | 0.5 (0.0 - 9.8) | 518.7 (0 - 4,331.3) | 1,303.3 (2.9 - 2,526.6) | 123.3 (0.0 - 930.5) |
| Eritrea | 1,251.4 (3.9 - 3,264.2) | 32.1 (2.3 - 239.3) | 1,983.5 (0 - 5,552.3) | 2,123.7 (2.7 - 4,505.7) | 847.2 (5.1 - 2,630.9) |
| Ethiopia | 1,844.7 (17.4 - 4,195.8) | 13.8 (0.3 - 80.1) | 2,761.8 (0 - 6,709.6) | 2,616.7 (4.1 - 4,979.0) | 1,552.0 (27.8 - 3,903.3) |
| Gabon | 1,089.6 (19.2 - 3,384.0) | 92.9 (14.0 - 389.1) | 1,535.3 (0 - 5,185.7) | 1,448.7 (3.6 - 3,975.4) | 993.2 (27.7 - 3,208.2) |
| Ghana | 477.0 (14.9 - 1,712.9) | 654.0 (236.7 - 1,405.9) | 1,277.5 (0 - 5,361.5) | 737.2 (1.0 - 2,454.5) | 248.0 (1.7 - 948.3) |
| Guinea | 1,405.3 (6.8 - 2,992.3) | 86.5 (11.2 - 436.3) | 2,311.8 (0 - 5,638.1) | 1,641.7 (1.5 - 3,530.4) | 1,302.6 (9.8 - 2,609.2) |
| Gambia | 57.5 (0.0 - 344.5) | 3.9 (0.0 - 29.9) | 33.5 (0 - 460.5) | 178.3 (0.0 - 889.5) | 6.9 (0.0 - 85.1) |
| Guinea-Bissau | 59.9 (1.1 - 427.3) | 102.7 (17.0 - 414.5) | 50.2 (0 - 912.1) | 152.0 (0.0 - 914.6) | 14.4 (0.0 - 127.1) |
| Equatorial Guinea | 986.3 (10.2 - 3,981.5) | 93.3 (17.8 - 407.0) | 2,290.4 (0 - 5,834.8) | 1,512.6 (5.0 - 4,684.8) | 685.0 (13.0 - 3,805.8) |
| Kenya | 1,899.6 (88.1 - 4,266.2) | 920.1 (284.1 - 1,613.5) | 3,107.3 (0 - 7,726.2) | 1,669.3 (6.4 - 4,894.5) | 1,928.9 (118.2 - 3,756.9) |
| Liberia | 1,417.0 (115.5 - 3,185.3) | 3,407.4 (1,778.7 - 4,968.4) | 2,624.0 (0 - 5,946.8) | 1,167.8 (0.8 - 3,320.0) | 1,129.4 (8.1 - 2,495.8) |
| Lesotho | 93.2 (0.4 - 1,117.2) | 2.9 (0.0 - 41.4) | 145.2 (0 - 1,972.2) | 332.2 (0.3 - 2,474.0) | 17.2 (0.5 - 667.3) |
| Madagascar | 778.8 (5.4 - 1,951.9) | 66.4 (16.0 - 222.7) | 345.5 (0 - 2,664.6) | 577.3 (0.3 - 2,030.2) | 1,003.5 (7.4 - 1,992.4) |
| Mali | 722.5 (3.0 - 1,610.7) | 24.3 (0.4 - 174.3) | 864.3 (0 - 3,137.9) | 1,201.4 (1.5 - 2,443.8) | 531.3 (4.9 - 1,043.9) |
| Mozambique | 1,098.8 (66.1 - 3,003.3) | 2,024.4 (825.3 - 3,814.8) | 765.3 (0 - 3,735.8) | 898.8 (1.9 - 3,508.0) | 1,139.8 (13.2 - 2,519.8) |
| Malawi | 144.6 (0.8 - 1,077.9) | 67.0 (9.9 - 244.0) | 583.6 (0 - 4,397.7) | 182.9 (0.1 - 1,469.0) | 56.3 (0.3 - 386.5) |
| Namibia | 1,260.3 (35.8 - 3,997.0) | 3.6 (0.2 - 39.1) | 1,650.3 (0 - 5,082.7) | 2,555.3 (10.4 - 5,326.7) | 867.3 (52.4 - 3,744.2) |
| Niger | 2,223.7 (9.4 - 3,358.7) | 2.8 (0.0 - 50.4) | 1,742.6 (0 - 5,044.3) | 2,796.0 (3.4 - 4,261.0) | 2,372.9 (16.5 - 3,020.7) |
| Nigeria | 835.1 (25.6 - 1,876.7) | 667.6 (311.4 - 1,087.1) | 1,078.2 (0 - 4,913.3) | 530.5 (0.3 - 1,963.1) | 961.5 (6.2 - 1,400.7) |
| Rwanda | 97.7 (0.0 - 944.5) | 10.1 (0.3 - 48.6) | 463.9 (0 - 4,051.4) | 131.2 (0.0 - 1,396.2) | 39.6 (0.0 - 393.8) |
| Sudan | 1,827.9 (11.7 - 3,100.0) | 359.9 (55.8 - 752.7) | 2,575.3 (0 - 5,364.8) | 2,596.3 (2.1 - 4,146.2) | 1,541.5 (12.8 - 2,553.1) |
| Senegal | 1,007.6 (5.5 - 2,349.2) | 137.6 (36.0 - 470.7) | 1,429.9 (0 - 3,589.2) | 1,755.0 (4.0 - 3,663.4) | 688.0 (3.6 - 1,744.9) |
| Sierra Leone | 1,169.0 (26.3 - 2,763.8) | 731.7 (285.7 - 1,486.8) | 1,185.8 (0 - 4,964.6) | 862.3 (0.8 - 2,831.3) | 1,347.7 (14.4 - 2,541.0) |
| Somalia | 2,316.8 (15.4 - 4,060.3) | 336.0 (82.6 - 884.3) | 3,629.2 (0 - 7,189.2) | 3,535.3 (5.9 - 5,261.9) | 1,691.8 (13.2 - 3,252.1) |
| South Sudan | 2,957.8 (20.8 - 4,245.2) | 737.5 (91.4 - 1,884.3) | 3,385.8 (0 - 5,779.9) | 4,054.2 (4.4 - 5,090.8) | 2,564.5 (24.9 - 3,785.9) |
| Swaziland | 287.1 (4.3 - 2,387.3) | 66.4 (10.4 - 252.9) | 453.8 (0 - 4,302.7) | 412.8 (0.9 - 3,301.8) | 237.4 (5.6 - 1,983.0) |
| Chad | 2,162.7 (13.1 - 3,332.1) | 206.8 (53.9 - 729.9) | 2,342.1 (0 - 4,854.8) | 2,776.3 (3.4 - 4,095.4) | 2,091.7 (14.8 - 2,989.5) |
| Togo | 1,338.5 (69.4 - 2,903.5) | 1,824.8 (987.1 - 3,068.7) | 2,196.8 (0 - 6,136.8) | 1,559.2 (2.2 - 3,563.6) | 1,058.6 (10.7 - 2,103.7) |
| Tanzania | 584.2 (7.2 - 2,452.0) | 456.0 (52.6 - 1,028.0) | 925.7 (0 - 4,624.8) | 700.2 (1.3 - 3,343.7) | 482.3 (5.5 - 1,805.9) |
| Uganda | 412.6 (1.8 - 1,746.5) | 74.7 (11.5 - 293.5) | 1,575.4 (0 - 5,197.2) | 419.6 (0.3 - 2,283.1) | 227.9 (1.6 - 968.2) |
| South Africa | 171.7 (1.2 - 1,654.3) | 20.5 (3.0 - 99.4) | 240.9 (0 - 2,471.5) | 411.6 (0.4 - 2,807.5) | 112.1 (1.4 - 1,372.6) |
| Zambia | 478.3 (4.1 - 1,848.5) | 10.8 (1.5 - 55.2) | 666.5 (0 - 3,378.8) | 527.5 (0.5 - 2,302.3) | 478.3 (6.9 - 1,569.1) |
| Zimbabwe | 43.7 (0.6 - 591.2) | 40.6 (9.0 - 144.1) | 114.0 (0 - 1,496.1) | 88.9 (0.0 - 994.9) | 12.0 (0.0 - 308.7) |

CAR = Central African Republic; DR Congo = Democratic Republic of the Congo

**Table E** in S1 Information. Estimated incidence rate of typhoid fever per 100,000 persons per year by Africa subregion, 2017

| Subregion | Current study | Mogasale[12] | Antillón [13] | Stanaway^*^[14] |
| --- | --- | --- | --- | --- |
| Eastern | 1,172.2 (23.2 - 2,956.6) | 537 | 620 (213 - 2,921) | 151.9 (132.0 - 174.6) |
| Middle | 1,278.2 (15.2 - 2,682.3) | 557 | 1,459 (371 - 6,984) | 81.4 (68.8 - 95.8) |
| Southern | 222.2 (2.7 - 1,776.1) | 170 | 149 (57 - 571) | 2.3 (2.0 - 2.6) |
| Western | 904.1 (22.9 - 1,989.9) | 160 | 753 (198 - 3075) | 161.1 (138.1 - 187.3) |

^*^includes paratyphoid fever.

**Table F** in S1 Information. Estimated number of cases by country and age group, sub-Saharan Africa, 2017

CAR = Central African Republic; Eq Guinea = Equatorial Guinea; DR Congo = Democratic Republic of the Congo

| Country | 0-1 y | 2-4 y | 5-14 y | over 14 y | Overall |
| --- | --- | --- | --- | --- | --- |
| Angola | 2,066 (483 - 8,453) | 44,014 (0 - 144,546) | 105,543 (292 - 285,577) | 354,298 (14,674 - 595,877) | 505,922 (15,449 - 1,034,453) |
| Burundi | 8 (0 - 85) | 12,018 (0 - 57,953) | 11,903 (0 - 61,166) | 20,102 (98 - 74,572) | 44,032 (98 - 193,775) |
| Benin | 24,469 (9,683 - 38,053) | 24,181 (0 - 57,001) | 63,425 (86 - 113,945) | 137,902 (1,397 - 184,643) | 249,976 (11,166 - 393,642) |
| Burkina Faso | 191 (32 - 891) | 3,496 (0 - 36,665) | 15,353 (2 - 58,486) | 1,643 (5 - 13,084) | 20,682 (39 - 109,126) |
| Botswana | 0 (0 - 1) | 661 (0 - 6,070) | 7,524 (25 - 22,835) | 3,444 (185 - 33,506) | 11,628 (210 - 62,412) |
| CAR | 1,859 (313 - 4,899) | 14,489 (0 - 28,065) | 41,816 (34 - 71,830) | 60,702 (466 - 93,704) | 118,866 (814 - 198,498) |
| Cote d'Ivoire | 5,788 (2,080 - 12,407) | 17,209 (0 - 79,526) | 43,533 (42 - 156,236) | 63,028 (421 - 165,599) | 129,558 (2,543 - 413,767) |
| Cameroon | 1,646 (177 - 8,866) | 27,429 (0 - 106,983) | 56,558 (63 - 186,964) | 82,220 (935 - 233,556) | 167,853 (1,175 - 536,369) |
| DR Congo | 10,240 (1,552 - 38,515) | 115,955 (0 - 384,301) | 305,575 (230 - 784,503) | 720,449 (5,444 - 1,197,770) | 1,152,219 (7,225 - 2,405,090) |
| Congo | 803 (153 - 2,034) | 6,347 (0 - 32,309) | 15,017 (20 - 40,727) | 14,946 (338 - 39,786) | 37,114 (511 - 114,856) |
| Djibouti | 0 (0 - 1) | 45 (0 - 372) | 373 (1 - 724) | 109 (0 - 822) | 527 (1 - 1,919) |
| Eritrea | 73 (5 - 546) | 6,824 (0 - 19,103) | 23,443 (30 - 49,738) | 19,624 (119 - 60,940) | 49,965 (154 - 130,327) |
| Ethiopia | 827 (20 - 4,805) | 231,862 (0 - 563,289) | 669,890 (1,056 - 1,274,678) | 873,975 (15,652 - 2,198,030) | 1,776,554 (16,728 - 4,040,803) |
| Gabon | 129 (20 - 542) | 3,085 (0 - 10,421) | 7,980 (20 - 21,899) | 15,309 (427 - 49,453) | 26,504 (467 - 82,315) |
| Ghana | 10,166 (3,680 - 21,855) | 29,289 (0 - 122,919) | 48,378 (68 - 161,076) | 41,377 (284 - 158,196) | 129,210 (4,032 - 464,046) |
| Guinea | 659 (85 - 3,323) | 24,384 (0 - 59,471) | 48,954 (45 - 105,275) | 83,267 (626 - 166,791) | 157,265 (756 - 334,860) |
| Gambia | 3 (0 - 22) | 36 (0 - 495) | 538 (0 - 2,684) | 41 (0 - 504) | 618 (0 - 3,705) |
| Guinea-Bissau | 131 (22 - 529) | 93 (0 - 1,692) | 814 (0 - 4,894) | 168 (0 - 1,478) | 1,206 (22 - 8,594) |
| Equatorial Guinea | 18 (3 - 78) | 620 (0 - 1,580) | 1,116 (4 - 3,455) | 1,294 (25 - 7,190) | 3,048 (32 - 12,304) |
| Kenya | 25,487 (7,870 - 44,692) | 125,131 (2 - 311,131) | 217,467 (833 - 637,617) | 559,371 (34,288 - 1,089,463) | 927,455 (42,992 - 2,082,902) |
| Liberia | 8,102 (4,229 - 11,814) | 9,100 (0 - 20,623) | 12,073 (9 - 34,322) | 24,911 (179 - 55,049) | 54,186 (4,417 - 121,807) |
| Lesotho | 2 (0 - 33) | 167 (0 - 2,269) | 1,143 (1 - 8,515) | 184 (5 - 7,118) | 1,497 (6 - 17,934) |
| Madagascar | 980 (236 - 3,288) | 7,207 (0 - 55,588) | 35,972 (18 - 126,494) | 143,377 (1,054 - 284,658) | 187,536 (1,308 - 470,028) |
| Mali | 388 (6 - 2,775) | 18,761 (0 - 68,112) | 69,874 (87 - 142,130) | 55,055 (504 - 108,172) | 144,077 (597 - 321,189) |
| Mozambique | 38,515 (15,702 - 72,577) | 20,406 (0 - 99,608) | 66,735 (145 - 260,466) | 170,768 (1,978 - 377,532) | 296,424 (17,824 - 810,183) |
| Malawi | 730 (108 - 2,656) | 9,312 (0 - 70,169) | 8,810 (4 - 70,752) | 5,107 (28 - 35,056) | 23,959 (140 - 178,633) |
| Namibia | 4 (0 - 49) | 2,968 (0 - 9,142) | 12,674 (52 - 26,420) | 12,233 (739 - 52,806) | 27,880 (791 - 88,418) |
| Niger | 50 (0 - 886) | 40,502 (0 - 117,241) | 168,646 (207 - 257,012) | 249,199 (1,735 - 317,232) | 458,397 (1,942 - 692,371) |
| Nigeria | 91,991 (42,916 - 149,797) | 206,394 (0 - 940,527) | 280,397 (182 - 1,037,518) | 1,046,750 (6,803 - 1,524,918) | 1,625,532 (49,901 - 3,652,760) |
| Rwanda | 74 (3 - 355) | 4,818 (0 - 42,079) | 4,183 (0 - 44,527) | 2,881 (0 - 28,686) | 11,956 (3 - 115,647) |
| Sudan | 8,822 (1,369 - 18,450) | 86,989 (0 - 181,212) | 243,445 (199 - 388,771) | 329,981 (2,732 - 546,536) | 669,237 (4,300 - 1,134,968) |
| Senegal | 1,106 (289 - 3,783) | 16,602 (0 - 41,674) | 57,487 (130 - 119,998) | 48,210 (254 - 122,266) | 123,406 (673 - 287,720) |
| Sierra Leone | 2,357 (921 - 4,790) | 5,432 (0 - 22,742) | 11,794 (11 - 38,728) | 41,670 (445 - 78,563) | 61,254 (1,377 - 144,823) |
| Somalia | 2,520 (620 - 6,634) | 36,215 (0 - 71,739) | 91,428 (152 - 136,083) | 80,536 (630 - 154,812) | 210,700 (1,402 - 369,268) |
| South Sudan | 6,561 (813 - 16,764) | 47,740 (0 - 81,498) | 155,288 (167 - 194,990) | 184,357 (1,787 - 272,167) | 393,947 (2,767 - 565,418) |
| Swaziland | 38 (6 - 145) | 387 (0 - 3,667) | 1,097 (2 - 8,774) | 1,669 (39 - 13,942) | 3,190 (48 - 26,528) |
| Chad | 2,287 (596 - 8,071) | 35,395 (0 - 73,370) | 108,069 (131 - 159,415) | 146,177 (1,035 - 208,921) | 291,927 (1,762 - 449,778) |
| Togo | 8,383 (4,535 - 14,098) | 14,306 (0 - 39,964) | 29,200 (40 - 66,737) | 45,238 (458 - 89,894) | 97,127 (5,034 - 210,693) |
| Tanzania | 16,374 (1,890 - 36,912) | 45,688 (0 - 228,267) | 97,174 (182 - 464,030) | 134,246 (1,537 - 502,702) | 293,482 (3,609 - 1,231,912) |
| Uganda | 2,071 (320 - 8,142) | 62,157 (0 - 205,054) | 47,960 (32 - 260,976) | 46,039 (322 - 195,555) | 158,227 (674 - 669,728) |
| South Africa | 459 (68 - 2,221) | 8,240 (0 - 84,521) | 42,653 (38 - 290,916) | 44,835 (550 - 549,211) | 96,187 (656 - 926,870) |
| Zambia | 124 (18 - 635) | 10,917 (0 - 55,348) | 24,712 (24 - 107,856) | 43,943 (636 - 144,165) | 79,696 (677 - 308,004) |
| Zimbabwe | 351 (78 - 1,246) | 1,460 (0 - 19,167) | 3,278 (0 - 36,690) | 960 (1 - 24,714) | 6,050 (78 - 81,817) |

**Table G** in S1 Information. Estimated number of cases by Africa subregion, 2017 (unit = thousands)

|  | Current study | Kim [16] | Antillón [17] | Stanaway[18] |
| --- | --- | --- | --- | --- |
| Eastern Africa | 4,461 (88 to 11,250) | 1,136.5 (923.3 to 1,408.9) | 2,400 (800 to 11,300) | 726.4 (615.6 to 856.5) |
| Middle Africa | 2,303 (27 to 4,834) | 713.5 (569.0 to 889.6) | 1,700 (400 to 8,400) | 119.2 (97.2 to 145.1) |
| Southern Africa | 140 (2 to 1,122) | 103.5 (75.8 to 140.9) | 100 (40 to 400) | 1.7 (1.5 to 2.0) |
| Western Africa | 3,252 (82 to 7,159) | 1,290.1 (1,033.7 to 1,609.7) | 2,800 (700, 11,200) | 653.2 (534.8 to 794.0) |

**Table H** in S1 Information. Comparison of incidence rates per 100, 000 person-years by country

| Country | Current study | Stanaway[18] | Antillón[17] | Mogasale | Kim[16] |
| --- | --- | --- | --- | --- | --- |
| Angola | 2,050.4 (62.6 - 4,192.5) | 38.9 (24.0 - 59.4) | 287.1 | 586.2 | 586.2 |
| Burundi | 445.2 (1.0 - 1,959.2) | 117.2 (73.7 - 174.8) | 1,980.6 | 439.4 | 273.6 |
| Benin | 2,320.3 (103.6 - 3,653.8) | 128.5 (80.4 - 198.3) | 1,925.8 | 155.6 | 410.9 |
| Burkina Faso | 100.3 (0.2 - 529.1) | 390.1 (276.2 - 533.4) | 570.8 | 143.7 | 518.2 |
| Botswana | 523.4 (9.5 - 2,809.3) | 1.8 (1.2 - 2.6) | 128.9 | 175.6 | 175.6 |
| CAR | 1,928.0 (13.2 - 3,219.7) | 40.1 (24.9 - 60.6) | 843.4 | 529.5 | 529.5 |
| Cote d'Ivoire | 557.6 (10.9 - 1,780.8) | 121.3 (76.6 - 183.5) | 607.1 | 138.5 | 366.3 |
| Cameroon | 653.7 (4.6 - 2,088.9) | 127.5 (79.9 - 192.0) | 264.5 | 462.8 | 462.8 |
| DR Congo | 1,189.3 (7.5 - 2,482.5) | 38.8 (23.9 - 59.1) | 1,622.9 | 584.0 | 584.0 |
| Congo | 351.3 (4.8 - 1,087.1) | 33.0 (20.3 - 51.0) | 365.1 | 493.4 | 493.4 |
| Djibouti | 400.8 (0.6 - 1,459.6) | 96.7 (61.9 - 143.2) | 104.5 | 375.0 | 232.8 |
| Eritrea | 1,251.4 (3.9 - 3,264.2) | 109.6 (69.8 - 161.2) | 516.5 | 523.3 | 326.6 |
| Ethiopia | 1,844.7 (17.4 - 4,195.8) | 137.9 (85.3 - 211.4) | 613.7 | 629.9 | 392.4 |
| Gabon | 1,089.6 (19.2 - 3,384.0) | 28.5 (18.1 - 43.3) | 221.3 | 407.6 | 407.6 |
| Ghana | 477.0 (14.9 - 1,712.9) | 229.9 (163.3 - 331.6) | 205.8 | 124.4 | 250.2 |
| Guinea | 1,405.3 (6.8 - 2,992.3) | 135.5 (85.0 - 201.4) | 649.1 | 140.0 | 369.8 |
| Gambia | 57.5 (0.0 - 344.5) | 125.2 (80.0 - 187.7) | 199.0 | 127.3 | 336.2 |
| Guinea-Bissau | 59.9 (1.1 - 427.3) | 22.7 (15.2 - 33.1) | 269.0 | 165.4 | 20.1 |
| Equatorial Guinea | 986.3 (10.2 - 3,981.5) | 35.7 (22.1 - 53.9) | 12.4 | NA | NA |
| Kenya | 1,899.6 (88.1 - 4,266.2) | 274.2 (170.5 - 412.0) | 193.6 | 809.4 | 630.5 |
| Liberia | 1,417.0 (115.5 - 3,185.3) | 135.3 (82.4 - 209.8) | 1,337.7 | 142.1 | 375.3 |
| Lesotho | 93.2 (0.4 - 1,117.2) | 2.3 (1.5 - 3.3) | 47.7 | 204.7 | 204.7 |
| Madagascar | 778.8 (5.4 - 1,951.9) | 85.7 (59.5 - 118.4) | 1,252.9 | 614.3 | 82.1 |
| Mali | 722.5 (3.0 - 1,610.7) | 137.0 (83.9 - 211.2) | 408.2 | 160.7 | 424.1 |
| Mozambique | 1,098.8 (66.1 - 3,003.3) | 115.3 (73.8 - 171.6) | 293.6 | 658.9 | 410.5 |
| Malawi | 144.6 (0.8 - 1,077.9) | 33.8 (26.8 - 42.7) | 174.3 | 478.9 | 298.2 |
| Namibia | 1,260.3 (35.8 - 3,997.0) | 2.0 (1.4 - 3.0) | 158.7 | 179.3 | 179.3 |
| Niger | 2,223.7 (9.4 - 3,358.7) | 144.5 (88.5 - 221.0) | 453.9 | 193.8 | 510.3 |
| Nigeria | 835.1 (25.6 - 1,876.7) | 146.7 (89.4 - 227.9) | 605.4 | 170.8 | 451.4 |
| Rwanda | 97.7 (0.0 - 944.5) | 104.6 (64.8 - 157.9) | 344.9 | 506.3 | 316.4 |
| Sudan | 1,827.9 (11.7 - 3,100.0) | NA | 254.4 | 24.6 | 24.6 |
| Senegal | 1,007.6 (5.5 - 2,349.2) | 113.1 (72.2 - 169.0) | 115.2 | 132.5 | 349.8 |
| Sierra Leone | 1,169.0 (26.3 - 2,763.8) | 134.0 (84.0 - 201.0) | 925.1 | 188.1 | 496.6 |
| Somalia | 2,316.8 (15.4 - 4,060.3) | 116.0 (71.8 - 172.6) | 481.8 | 701.3 | 438.1 |
| South Sudan | 2,957.8 (20.8 - 4,245.2) | 121.6 (76.0 - 178.2) | 259.9 | NA | NA |
| Swaziland | 287.1 (4.3 - 2,387.3) | 2.2 (1.4 - 3.3) | 116.1 | 243.5 | 243.5 |
| Chad | 2,162.7 (13.1 - 3,332.1) | 145.8 (89.4 - 224.3) | 904.5 | 560.8 | 560.8 |
| Togo | 1,338.5 (69.4 - 2,903.5) | 129.9 (82.6 - 195.3) | 168.1 | 156.1 | 413 |
| Tanzania | 584.2 (7.2 - 2,452.0) | 151.5 (104.7 - 207.2) | 553.3 | 69.0 | 164.7 |
| Uganda | 412.6 (1.8 - 1,746.5) | 110.0 (68.6 - 164.2) | 146.1 | 527.0 | 329.4 |
| South Africa | 171.7 (1.2 - 1,654.3) | 1.8 (1.2 - 2.6) | 72.5 | 176.1 | 176.1 |
| Zambia | 478.3 (4.1 - 1,848.5) | 106.8 (66.2 - 161.1) | 362 | 588.2 | 367 |
| Zimbabwe | 43.7 (0.6 - 591.2) | 2.4 (1.6 - 3.5) | 274.4 | 403.4 | 250.3 |

**Fig T** in S1 Information. Incidence rate estimates per 100,000 person-years by country. (A) shows existing incidence rate estimates at the country level. The upper bounds of some estimates by Antillón *et* *al*. and in the current study go over 4,000. (B) highlights the differences between the estimates by Antillón *et* *al*. and by this study that take a similar approach of using grid-level geospatial covariates.

(A)

**
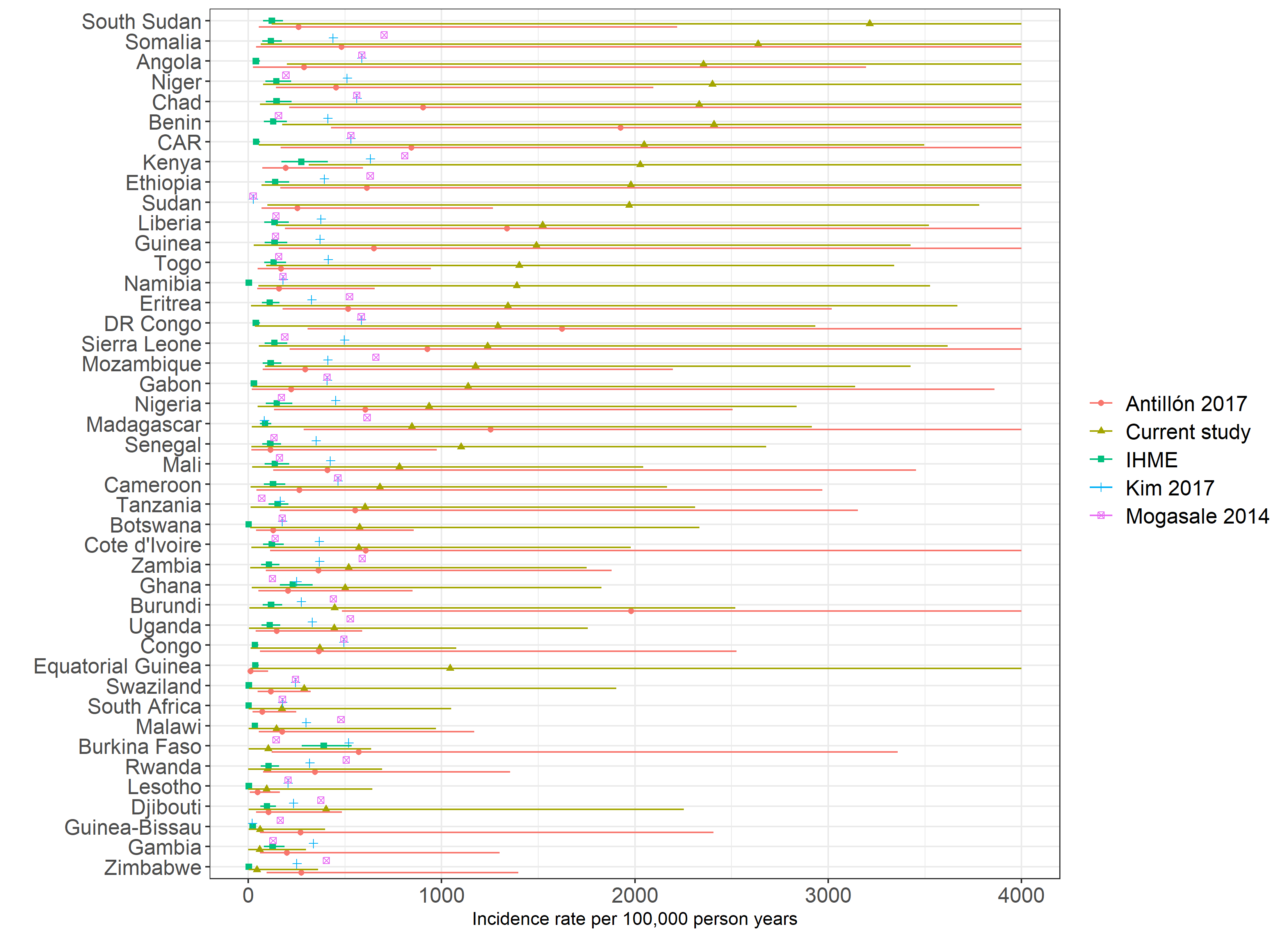
**

(B)

**
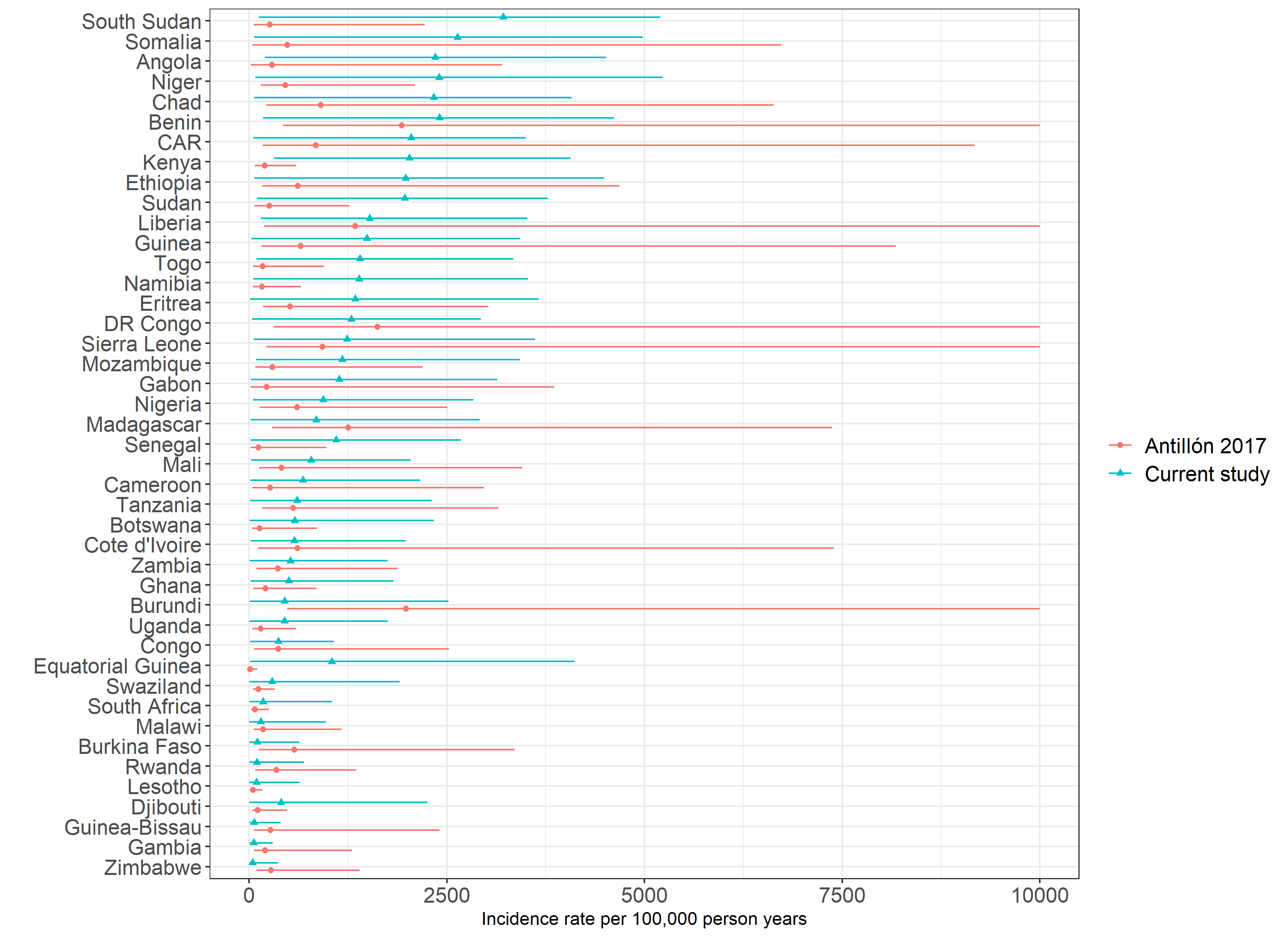
**

IHME = Institute for Health Metrics and Evaluation. Incidence rates were downloaded from the web portal (<https://vizhub.healthdata.org/gbd-results/>) for the year 2017. The estimates include both typhoid and paratyphoid fever.

**References**

1. Breiman RF, Cosmas L, Njuguna H, Audi A, Olack B, Ochieng JB, et al. Population-based incidence of typhoid fever in an urban informal settlement and a rural area in Kenya: implications for typhoid vaccine use in Africa. PLoS One. 2012;7: e29119. doi:10.1371/journal.pone.0029119

2. Marks F, von Kalckreuth V, Aaby P, Adu-Sarkodie Y, El Tayeb MA, Ali M, et al. Incidence of invasive salmonella disease in sub-Saharan Africa: a multicentre population-based surveillance study. Lancet Glob Health. 2017;5: e310–e323. doi:10.1016/S2214-109X(17)30022-0

3. Marks F, Im J, Park SE, Pak G, Jeon H, Wandji Nana LR, et al. The Severe Typhoid in Africa Program: Incidences Of Typhoid Fever in Burkina Faso, Democratic Republic of Congo, Ethiopia, Ghana, Madagascar, And Nigeria. Rochester, NY; 2022. doi:10.2139/ssrn.4292849

4. Meiring JE, Shakya M, Khanam F, Voysey M, Phillips MT, Tonks S, et al. Burden of enteric fever at three urban sites in Africa and Asia: a multicentre population-based study. Lancet Glob Health. 2021;9: e1688–e1696. doi:10.1016/S2214-109X(21)00370-3

5. Thriemer K, Ley B, Ame S, von Seidlein L, Pak GD, Chang NY, et al. The burden of invasive bacterial infections in Pemba, Zanzibar. PLoS One. 2012;7: e30350. doi:10.1371/journal.pone.0030350

6. Park SE, Toy T, Cruz Espinoza LM, Panzner U, Mogeni OD, Im J, et al. The Severe Typhoid Fever in Africa Program: Study Design and Methodology to Assess Disease Severity, Host Immunity, and Carriage Associated With Invasive Salmonellosis. Clin Infect Dis. 2019;69: S422–S434. doi:10.1093/cid/ciz715

7. Deshpande A, Miller-Petrie MK, Lindstedt PA, Baumann MM, Johnson KB, Blacker BF, et al. Mapping geographical inequalities in access to drinking water and sanitation facilities in low-income and middle-income countries, 2000–17. The Lancet Global Health. 2020;8: e1162–e1185. doi:10.1016/S2214-109X(20)30278-3

8. Copernicus Climate Change Service. Temperature and precipitation gridded data for global and regional domains derived from in-situ and satellite observations. ECMWF; 2021. doi:10.24381/CDS.11DEDF0C

9. Kinyoki DK, Osgood-Zimmerman AE, Pickering BV, Schaeffer LE, Marczak LB, Lazzar-Atwood A, et al. Mapping child growth failure across low- and middle-income countries. Nature. 2020;577: 231–234. doi:10.1038/s41586-019-1878-8

10. Dwyer-Lindgren L, Cork MA, Sligar A, Steuben KM, Wilson KF, Provost NR, et al. Mapping HIV prevalence in sub-Saharan Africa between 2000 and 2017. Nature. 2019;570: 189–193. doi:10.1038/s41586-019-1200-9

11. Weiss DJ, Nelson A, Gibson HS, Temperley W, Peedell S, Lieber A, et al. A global map of travel time to cities to assess inequalities in accessibility in 2015. Nature. 2018;553: 333–336. doi:10.1038/nature25181

12. Carrea L, Embury O, Merchant CJ. Datasets related to in-land water for limnology and remote sensing applications: distance-to-land, distance-to-water, water-body identifier and lake-centre co-ordinates. Geoscience Data Journal. 2015;2: 83–97. doi:10.1002/gdj3.32

13. Hollister J, Shah T, Robitaille AL, Beck MW, Johnson M. elevatr: Access Elevation Data from Various APIs. 2021. doi:10.5281/zenodo.5809645

14. Lloyd CT, Sorichetta A, Tatem AJ. High resolution global gridded data for use in population studies. Sci Data. 2017;4: 170001. doi:10.1038/sdata.2017.1

15. Arlot S, Celisse A. A survey of cross-validation procedures for model selection. Statistics Surveys. 2010;4: 40–79. doi:10.1214/09-SS054

16. Kim J-H, Mogasale V, Im J, Ramani E, Marks F. Updated estimates of typhoid fever burden in sub-Saharan Africa. Lancet Glob Health. 2017;5: e969. doi:10.1016/S2214-109X(17)30328-5

17. Antillón M, Warren JL, Crawford FW, Weinberger DM, Kürüm E, Pak GD, et al. The burden of typhoid fever in low- and middle-income countries: A meta-regression approach. PLoS Negl Trop Dis. 2017;11: e0005376. doi:10.1371/journal.pntd.0005376

18. Stanaway JD, Reiner RC, Blacker BF, Goldberg EM, Khalil IA, Troeger CE, et al. The global burden of typhoid and paratyphoid fevers: a systematic analysis for the Global Burden of Disease Study 2017. Lancet Infect Dis. 2019;19: 369–381. doi:10.1016/S1473-3099(18)30685-6
